# *Wolbachia* mediates crosstalk between miRNA and Toll pathways to enhance resistance to dengue virus in *Aedes aegypti*

**DOI:** 10.1371/journal.ppat.1012296

**Published:** 2024-06-17

**Authors:** Lingzhi She, Mengyi Shi, Ting Cao, Hao Yuan, Renke Wang, Weifeng Wang, Yueting She, Chaojun Wang, Qin Zeng, Wei Mao, Yalan Zhang, Yong Wang, Zhiyong Xi, Xiaoling Pan

**Affiliations:** 1 The Engineering Research Center of Reproduction and Translational Medicine of Hunan Province, Department of Medical Laboratory Science, Hunan Normal University School of Medicine, Changsha, Hunan, P.R. China; 2 The Key Laboratory of Protein Chemistry and Developmental Biology of Fish of the Ministry of Education, Hunan Normal University, Changsha, Hunan, P.R. China; 3 Hunan Provincial Center for Disease Control and Prevention, Changsha, Hunan, P.R. China; 4 Changsha City Center for Disease Control and Prevention, Changsha, Hunan, P.R. China; 5 Department of Forensic Science, School of Basic Medical Sciences, Central South University, Changsha, Hunan, P.R. China; 6 Department of Microbiology, Genetics, & Immunology, Michigan State University, East Lansing, Michigan, United States of America; UNC-Chapel Hill: The University of North Carolina at Chapel Hill, UNITED STATES

## Abstract

The obligate endosymbiont *Wolbachia* induces pathogen interference in the primary disease vector *Aedes aegypti*, facilitating the utilization of *Wolbachia*-based mosquito control for arbovirus prevention, particularly against dengue virus (DENV). However, the mechanisms underlying *Wolbachia*-mediated virus blockade have not been fully elucidated. Here, we report that *Wolbachia* activates the host cytoplasmic miRNA biogenesis pathway to suppress DENV infection. Through the suppression of the long noncoding RNA aae-lnc-2268 by *Wolbachia w*AlbB, aae-miR-34-3p, a miRNA upregulated by the *Wolbachia* strains *w*AlbB and *w*MelPop, promoted the expression of the antiviral effector *defensin* and *cecropin* genes through the Toll pathway regulator *MyD88*. Notably, anti-DENV resistance induced by *Wolbachia* can be further enhanced, with the potential to achieve complete virus blockade by increasing the expression of aae-miR-34-3p in *Ae*. *aegypti*. Furthermore, the downregulation of aae-miR-34-3p compromised *Wolbachia*-mediated virus blockade. These findings reveal a novel mechanism by which *Wolbachia* establishes crosstalk between the cytoplasmic miRNA pathway and the Toll pathway via aae-miR-34-3p to strengthen antiviral immune responses against DENV. Our results will aid in the advancement of *Wolbachia* for arbovirus control by enhancing its virus-blocking efficiency.

## Author summary

Although the involvement of miRNAs has been reported in the symbiosis of *Wolbachia* in *Ae*. *aegypti*, the role of miRNAs in the significant strong virus inhibition by *Wolbachia w*AlbB is poorly understood. To our knowledge, we illustrated a novel miRNA-based antiviral mechanism involved in *Wolbachia*-mediated viral interference according to the first global analysis of miRNA differential expression triggered by *Wolbachia w*AlbB. Our findings could improve the understanding of miRNA functions involved in interactions among symbiotic bacteria, arboviruses, and mosquito hosts. In particular, we identified an antiviral miRNA that could enhance the *Wolbachia*-mediated antiviral immune response in *Ae*. *aegypti*, suggesting that this novel molecule could improve mosquito control techniques to combat the current resurgence of dengue and other arbovirus diseases.

## Introduction

The global surge in dengue incidence across various geographic regions in recent decades has escalated the worldwide challenges associated with dengue epidemics [1]. However, due to the lack of specific therapeutics and effective vaccines for use against all serotypes of dengue virus (DENV) [2], vector control has remained the key strategy in dengue prevention and control. As a prominent innovative strategy, *Wolbachia*-based mosquito control techniques have been recommended for dengue prevention by the World Health Organization (WHO). An important theoretical basis for this technique is that the intracellular endosymbiotic bacterium *Wolbachia* can confer on hosts, such as *Aedes aegypti* [3] and *Aedes albopictus* [4], broad-spectrum resistance against human pathogens such as DENV, yellow fever virus, chikungunya virus and *Plasmodium* [4–7]. By exploring the underlying mechanism of *Wolbachia*-mediated pathogen interference, studies have found that the *Wolbachia*-activated Toll pathway plays an essential role in the antiviral immune response in the primary dengue vector *Ae*. *aegypti* [8–10]. Although we have demonstrated that *Wolbachia* induces reactive oxygen species (ROS)-dependent activation of the Toll pathway to control DENV in *Ae*. *aegypti* [8], a single antiviral mechanism cannot fully explain the robust *Wolbachia*-induced inhibition or even complete blockage of DENV transmission in *Ae*. *aegypti*. An in-depth understanding of multiple antiviral mechanisms induced by *Wolbachia* is important for practical utilization of *Wolbachia* for mosquito vector control strategies.

There is growing evidence suggesting that microRNAs (miRNAs) function in *Wolbachia* symbiosis [11] and are positively associated with *Wolbachia*-mediated pathogen interference in *Aedes* mosquitoes [5,12]. Hence, the communication between *Wolbachia* and host miRNA biological functions has received increasing attention. In *Ae*. *aegypti*, *w*MelPop, a strain of *Wolbachia* supergroup A identified from *Drosophila melanogaster* [13], triggers alterations in mosquito miRNA expression [11] and modifies the shuttling and structure of miRNA in cells [14]. This finding indicates that *Wolbachia* might manipulate *Ae*. *aegypti* miRNA biogenesis. Canonical miRNA biogenesis is a multistep process involving both the nucleus and cytoplasm that starts from the miRNA-encoding gene in the genome and progresses through post- or cotranscriptional processing of RNA polymerase II transcripts in the nucleus [15]. Subsequently, the primary miRNA (pri-miRNA) is cleaved by Drosha and DiGeorge syndrome critical region gene 8 (DGCR8)/Pasha to produce the precursor miRNA (pre-miRNA) [15]. After being exported into the cytoplasm, the pre-miRNA is processed into approximately 22-nucleotide (nt) molecules by Dicer-1 (DCR-1) and Argonaute-1 (AGO-1) [15]. DCR-1, a key component of the miRNA pathway, is an RNase III family protein that recognizes and processes pre-miRNA into short base-paired duplex miRNA and associates with AGO-1 for cytoplasmic maturation of miRNA [16]. Although the *Wolbachia* strain *w*MelPop has been shown to induce the production of *Ae*. *aegypti AGO-1* [14], little is known about the exact mechanism by which *Wolbachia* modulates the miRNA biogenesis pathway in response to DENV infection in *Ae*. *aegypti*. Elucidating the role of the miRNA biogenesis pathway in *Wolbachia*-mediated antiviral responses will deepen our comprehension of the various antiviral mechanisms employed by *Wolbachia* in its direct combat against DENV.

Viral infection assay data have shown that *Wolbachia* employs host miRNAs to inhibit DENV replication in *Ae*. *aegypti* cells through non-immune-priming antiviral activity [12,17]. The *Wolbachia* strain *w*MelPop induces the expression of aae-miR-2940 to inhibit DENV replication via nonimmune genes of the host, such as genes encoding DNA methyltransferase (*AaDnmt2*) [12] and the protein arginine methyltransferase 3 (*AaArgM3*) [17]. These miRNAs and mRNAs have been shown to function as regulators of *Wolbachia* growth density, and *Wolbachia* density is positively associated with the strength of the antiviral effect [5,18,19]. Aside from the involvement of the *Wolbachia* strain and its density in the pathogen interference, there are multiple mechanisms underlying the potent antiviral effect [20]. In particular, *Wolbachia w*AlbB, a *Wolbachia* supergroup B strain identified from *Ae*. *albopictus*, activates the Toll pathway, an important pathway that controls *Ae*. *aegypti* antiviral immune response [10] to promote the production of Cecropin and Defensin. Cecropin and Defensin are antimicrobial peptides (AMPs) with strong antiviral activity that play a crucial role in blocking DENV [8]. Consequently, the communication between *Wolbachia*-regulated miRNAs and the Toll pathway was investigated in our previous work. We have demonstrated that *Wolbachia* upregulates host long non-coding (lncRNA) to activate the Toll pathway and regulate intracellular ROS levels [21], and ROS are essential factors restricting DENV infection in mosquitoes [22,23]. These findings reveal that *Wolbachia* employs host non-coding RNAs as a novel player in the antiviral immune response of *Ae*. *aegypti*. However, the strength of the miRNA-based antiviral immune response in *Wolbachia*-mediated anti-DENV effects is still poorly understood. We speculate that the miRNAs coregulated by different *Wolbachia* strains might display greater potency in viral inhibition since the *Wolbachia* strain has been shown to be an important factor for viral inhibition. miRNAs with strong antiviral effects could be new molecular targets for improving the implementation of mosquito control techniques in the prevention and control of the current resurgence of dengue and other arboviral diseases.

In this study, we explored a novel antiviral mechanism, the crosstalk between miRNA and the Toll pathway, which was employed by *Wolbachia* to directly combat DENV in *Ae*. *aegypti*. We identified aae-miR-34-3p, an *Ae*. *aegypti* miRNA induced by two different strains of *Wolbachia* from supergroups A and B, which was negatively regulated by aae-lnc-2268, a long intergenic noncoding RNA (lincRNA) whose expression was suppressed by *Wolbachia*. Notably, aae-miR-34-3p enhanced the Toll pathway’s antiviral immune responses, particularly for *Cecropin* and *Defensin* family genes, by upregulating the expression of *MyD88*, a key regulator of the Toll pathway. Overall, we have demonstrated for the first time that the antiviral effect of aae-miR-34-3p plays a crucial role in *Wolbachia*-mediated DENV interference, achieved through crosstalk between the cytoplasmic miRNA biogenesis pathway and Toll pathway in *Ae*. *aegypti*.

## Results

### *Wolbachia* manipulates the cytoplasmic miRNA biogenesis pathway to suppress DENV replication in *Ae*. *aegypti*

**Fig 1:**
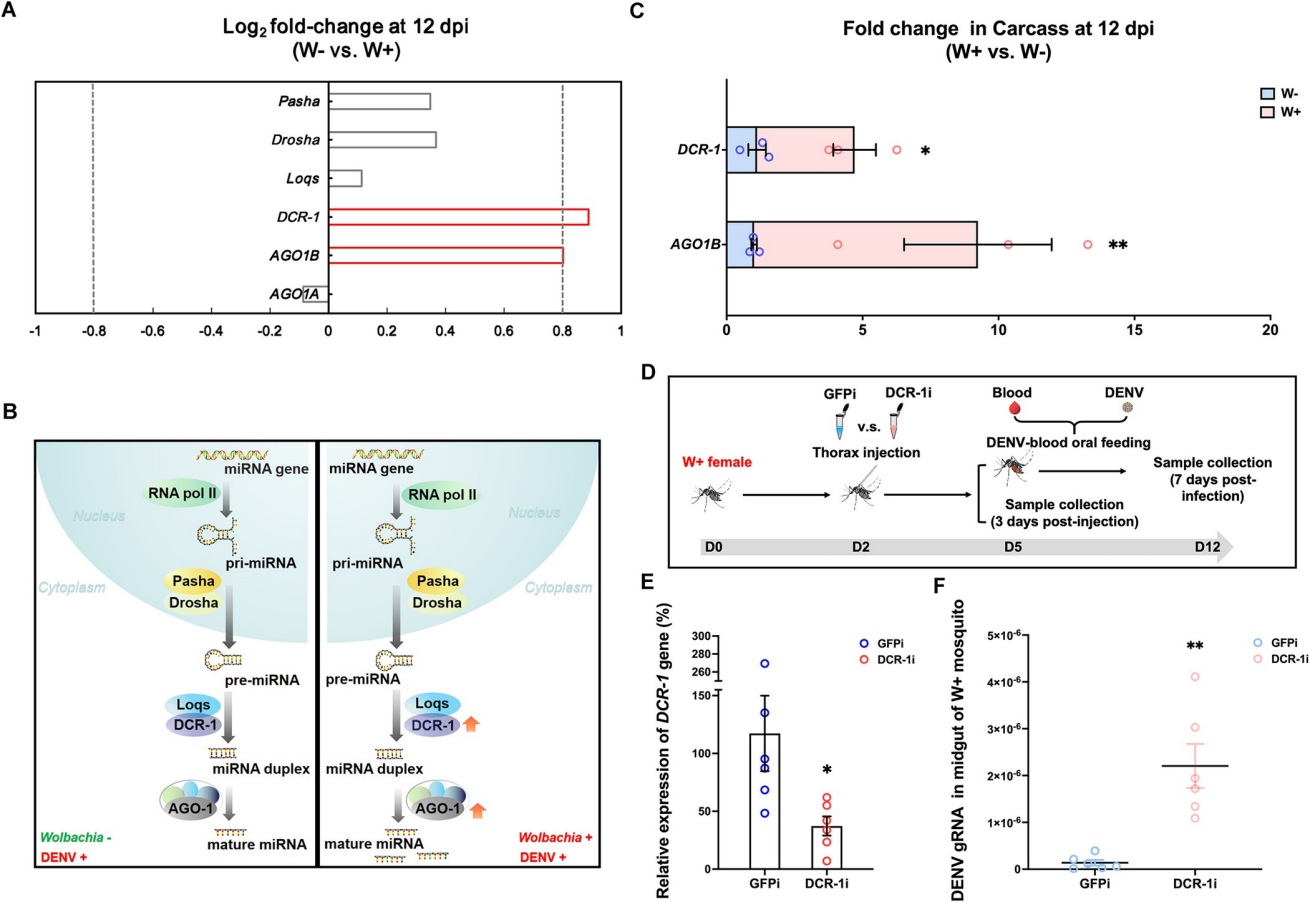
*Wolbachia w*AlbB activates the microRNA (miRNA) pathway to inhibit dengue virus (DENV) serotype 2 (DENV-2) replication in *Ae*. *aegypti*. (A) miRNA pathway alteration in DENV-2*-*infected *Ae*. *aegypti* triggered by *Wolbachia w*AlbB. The miRNA pathway regulator gene expression in the carcass (the mosquito tissue except the midgut) was compared between *Wolbachia w*AlbB-infected (W+, n = 4) and noninfected (W-, n = 4) female *Ae*. *aegypti* at 12 days post-DENV-2 infection (12 dpi) based on analysis of public microarray data [8]. The gray dotted line indicates the threshold value of log_2_(fold-change) >0.8 and log_2_(fold-change) <-0.8. (B) Schematic of the regulation of the miRNA biogenesis pathway by *Wolbachia w*AlbB in DENV-2*-*infected *Ae*. *aegypti*. The red arrow represents *Wolbachia w*AlbB-induced genes upon DENV infection. DENV+ indicates females with DENV infection, DENV- indicates females without DENV infection. (C) Quantitative PCR (qPCR) validation of *Wolbachia-*induced *Dcr-1* (two-sided t test, W+: n = 3, W-: n = 3, *P* = 0.0183) and *Ago1* (two-sided t test, W+: n = 3, W-: n = 3, *P* = 0.0048) gene expression microarray assays. (D) Schematic of the suppression of cytoplasmic miRNA biogenesis and viral infection in W+ mosquitoes. D0, D2, D5, and D12 indicate 0, 2, 5, and 12 days post-eclosion, respectively. (E) The knockdown efficiency of the *Dcr-1* gene in W+ female mosquitoes at 3 days post-dsRNA injection (two-sided t test, GFPi: n = 6, DCR-1i: n = 6, *P* = 0.0153). (F) DENV-2 infection level in dsRNA-treated W+ females at 7 days post-DENV-2 infection (two-sided t test, GFPi: n = 6, DCR-1i: n = 6, *P* = 0.0014). GFPi: W+ mosquitoes injected with GFP dsRNA, used as the control group. DCRi: W+ mosquitoes injected with dsRNA of the *Dcr* gene. The error bars indicate the standard error. The black horizontal line indicates the mean value of DENV infection level. Each circle indicates a replicate per group. **P < 0.01; *P < 0.05.

To explore the role of *Wolbachia* in the host miRNA biogenesis pathway during DENV infection in *Ae*. *aegypti*, we conducted a systematic comparative analysis of differential gene expression in the miRNA biogenesis pathway using the data from the midgut and carcass (mosquito tissue except the midgut) samples between *Wolbachia w*AlbB-infected (W+) and uninfected (W-) female *Ae*. *aegypti* 12 days post-DENV-2 infection (d.p.i.), which were retrieved from the microarray-based transcriptome profiles generated in our previous work [8]. The analysis results indicated that *Wolbachia* modulated cytoplasmic miRNA biogenesis in mosquito carcasses at 12 d.p.i. by elevating the expression of the *Dcr-1* and *Argonaute-1B* (*Ago-1B*) genes (Fig 1A and 1B), which encode key regulators of cytoplasmic miRNA biogenesis (Fig 1B). Furthermore, the induction of *Dcr-1* and *Ago-1B* gene expression by *w*AlbB in the carcass samples upon DENV-2 infection was confirmed via qPCR (Fig 1C).

To test the effect of *Wolbachia*-regulated cytoplasmic miRNA biogenesis on DENV replication in *Ae*. *aegypti*, suppression of cytoplasmic miRNA biogenesis was conducted via injection of double-stranded RNA (dsRNA) and then viral infection assays were conducted via oral feeding of a DENV-2-containing blood meal in W+ female mosquitoes (Fig 1D). Compared to the control females at 3 days post-dsGFP injection, the females with dsDCR1 injection showed a 62.75% decrease in the expression of *the Dcr-1* gene (Fig 1E). Strikingly, the suppression of cytoplasmic miRNA biogenesis via injection of dsDCR1 caused a marked 16-fold increase in the DENV RNA level in females at 7 days post-feeding of the DENV-2-containing blood meal (Fig 1F). These results illustrated that *Wolbachia* manipulated the host cytoplasmic miRNA biosynthesis pathway via *Dcr-1* upon DENV-2 infection, indicating that *Wolbachia*-induced cytoplasmic miRNA biosynthesis pathway may contribute to the inhibition of DENV-2 replication in *Ae*. *aegypti*.

### Both *Wolbachia w*AlbB and *w*MelPop regulate common miRNAs in *Ae*. *aegypti* cells

**Fig 2:**
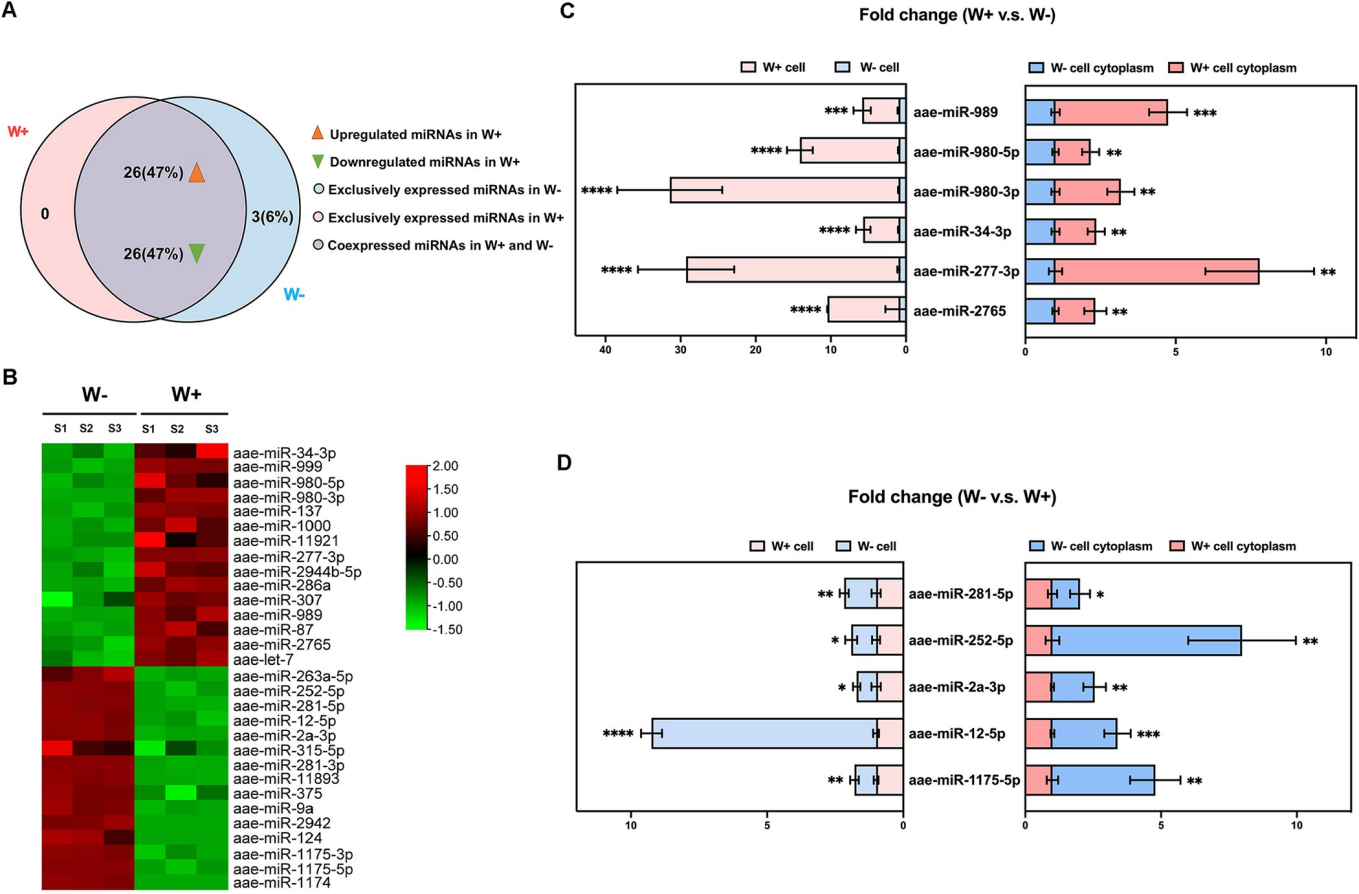
*Wolbachia w*AlbB triggers the differential expression of miRNAs in Aag2 *Ae*. *aegypti* cells. (A) Venn diagram of 55 DE miRNAs of *Ae*. *aegypti* induced by *Wolbachia w*AlbB according to small RNA sequencing. (B) Cluster analysis of the top 15 upregulated and downregulated DE miRNAs between W+ and W- cells with three replicates per cell identified by small RNA sequencing. According to the TPM value for DE miRNAs, red represents upregulated miRNAs, and green represents downregulated miRNAs. (C) The expression of 6 out of the top 15 up-regulated DE miRNAs was increased in whole-cell samples (left panel, two-sided t test, W-: n = 4, W+: n = 4, aae-miR-989: *P* = 0.0002, aae-miR-980-5p: *P* = 2.5421×10^−6^, aae-miR-980-3p: *P* = 9.9477×10^−6^, aae-miR-34-3p: *P* = 5.9804×10^−5^, aae-miR-277-3p: *P* = 1.6964×10^−5^, aae-miR-2765: *P* = 9.1317×10^−6^) and cytoplasm samples (right panel, two-sided t test, W-: n = 4, W+: n = 4, aae-miR-989: *P* = 0.0002, aae-miR-980-5p: *P* = 0.0052, aae-miR-980-3p: *P* = 0.0012, aae-miR-34-3p: *P* = 0.0023, aae-miR-277-3p: *P* = 0.0017, aae-miR-2765: *P* = 0.0058) from W+ cells in qPCR analysis. (D) qPCR analysis showed that the expression of 5 out of the top 15 downregulated DE miRNAs was decreased in whole-cell samples (left panel, two-sided t test, W-: n = 4, W+: n = 4, aae-miR-281-5p: *P* = 0.0036, aae-miR-252-5p: *P* = 0.0188, aae-miR-2a-3p: *P* = 0.0397, aae-miR-12-5p: *P* = 9.6512×10^−7^, aae-miR-1175-5p: *P* = 0.0043) and cytoplasm samples (right panel, two-sided t test, W-: n = 4, W+: n = 4, aae-miR-281-5p: *P* = 0.0291, aae-miR-252-5p: *P* = 0.0015, aae-miR-2a-3p: *P* = 0.0015, aae-miR-12-5p: *P* = 0.0006, aae-miR-1175-5p: *P* = 0.0015) from W+ cells in small RNA sequencing. *Actin* and *U6* were used as reference genes for normalization of cytoplasmic and nuclear miRNA quantification, respectively. Error bars indicate the standard error. ****P < 0.0001; ***P < 0.001; **P < 0.01; *P < 0.05.

To investigate the hypothesis that *Wolbachia w*AlbB might induce differentially expressed (DE) miRNAs through manipulation of the miRNA biogenesis pathway in *Ae*. *aegypti*, we performed small RNA sequencing on an Illumina NovaSeq 6000 platform to compare the host miRNA profiles between *Wolbachia w*AlbB-infected (W+) and uninfected (W-) *Ae*. *aegypti* Aag2 cells. According to the reference genome (GCF_002204515.2) and 164 mature miRNAs (AaegL1) of *Ae*. *aegypti*, 37,421,484 and 30,397,028 clean reads of 18–30 nt in length from W- and W+ cells, respectively, were used for miRNA identification via Bowtie software (S1 Table). As a result, we identified a total of 55 DE miRNAs out of 164 mature miRNAs with annotations in miRBase (https://www.mirbase.org/), including 26 upregulated miRNAs and 26 downregulated miRNAs in W+ cells (Fig 2A and S2 Table) as well as 3 miRNAs expressed exclusively in W- cells (Fig 2A and S2 Table).

To gain further insight into the impact of *Wolbachia w*AlbB on cytoplasmic miRNA biogenesis, the miRNA expression between W- and W+ cells was compared in both cytoplasm and whole cell using nucleocytoplasmic separation and qPCR. Among the top 15 induced or suppressed miRNAs identified by RNA sequencing analysis (Fig 2B), 6 upregulated miRNAs (aae-miR-989, aae-miR-980-5p, aae-miR-980-3p, aae-miR-34-3p, aae-miR-277-3p, and aae-miR-2765) and 5 downregulated miRNAs (aae-miR-281-5p, aae-miR-252-5p, aae-miR-2a-3p, aae-miR-12-5p, and aae-miR-1175-5p) were identified in both whole-cell samples and cytoplasm samples of W+ cells compared to W- cells (Figs 2C, S1, 2D and S2).These results indicate that *Wolbachia w*AlbB alters cytoplasmic miRNA levels through the miRNA biogenesis pathway in *Ae*. *aegypti*.

To explore the DE miRNAs regulated by different strains of *Wolbachia*, a comparative analysis of DE miRNAs induced by *Wolbachia* strains *w*AlbB and *w*MelPop was carried out using our miRNA data and currently available miRNA sequencing data from *Wolbachia* strain *w*MelPop-infected and noninfected *Ae*. *aegypti* cells [14]. Only 2 of the above 11 confirmed cytoplasmic DE miRNAs shared the same pattern of regulation by those both strains. Specifically, aae-miR-34-3p in the cytoplasm was upregulated, whereas aae-miR-1175-5p in the cytoplasm was downregulated, by both *w*AlbB and *w*MelPop. These results indicate a conserved patten in regulation of miRNA by different *Wolbachia* strains in *Ae*. *aegypti*.

### *Wolbachia* induces the expression of nuclear and cytoplasmic aae-miR-34-3p to enhance *MyD88* expression

We next tested the hypothesis that *Wolbachia* might employ cytoplasmic DE miRNAs to regulate the host antiviral Toll pathway in *Ae*. *aegypti*. The interaction between the above 2 cytoplasmic DE miRNAs, regulated commonly by both *w*AlbB and *w*MelPop, and Toll pathway regulator genes was predicted using RNAhybrid [24] and RNA22 [25] software. Only aae-miR-34-3p had a predicted potential interaction with the *MyD88* gene encoding the Toll pathway key regulator.

**Fig 3:**
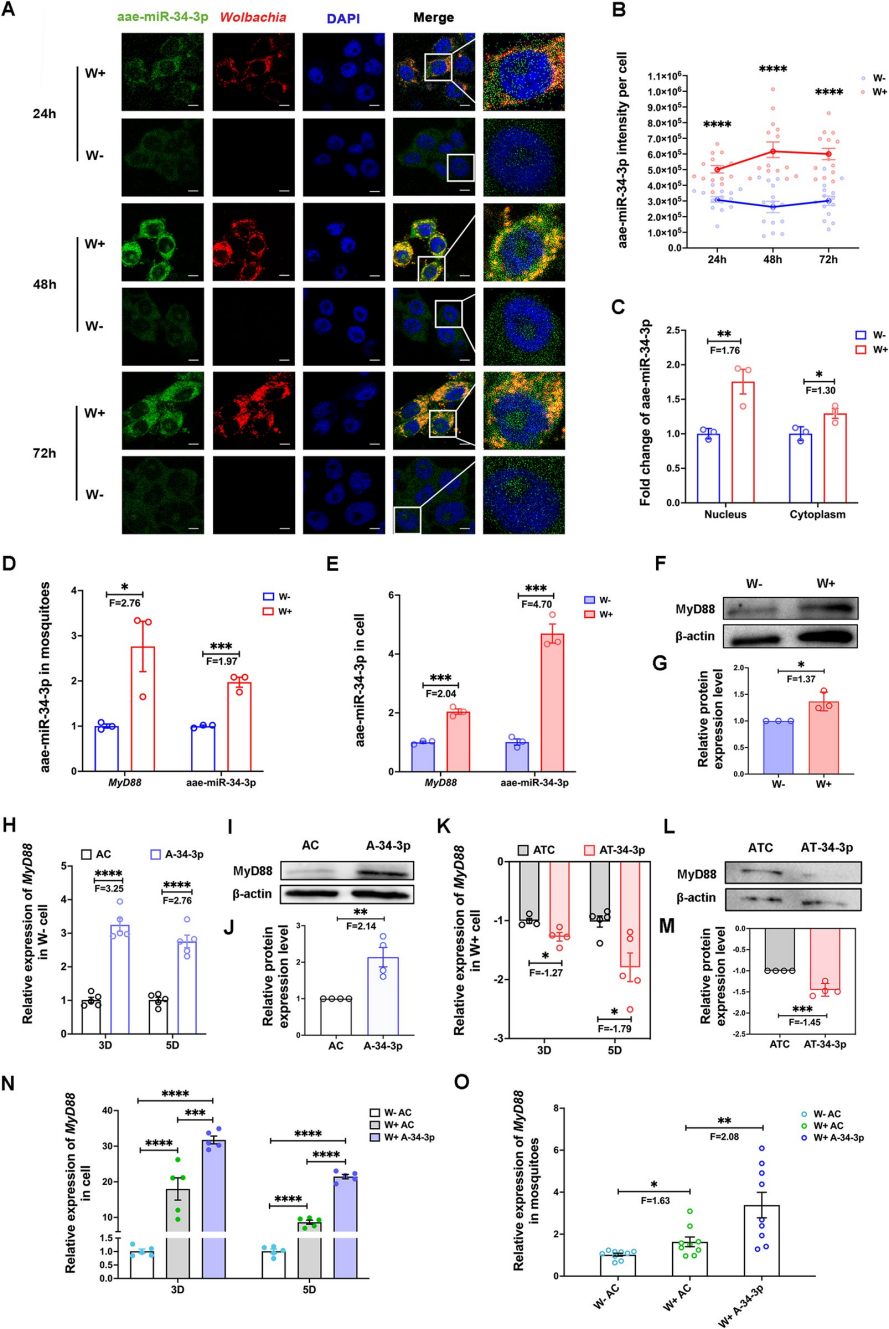
*Wolbachia w*AlbB induces aae-miR-34-3p expression to regulate *MyD88* gene expression in *Ae*. *aegypti*. (A) The subcellular distribution of aae-miR-34-3p and *Wolbachia w*AlbB in W- and W+ cells in an RNA-FISH assay. The white horizontal line represents 5 μm. (B) The fluorescence intensity of aae-miR-34-3p per cell in W- and W+ cells in an RNA-FISH assay at 24 h post-cell seeding (24 h, repeated measures ANOVA, W-: n = 14, W+: n = 15, *P* = 8.2527×10^−7^), 48 h post-cell seeding (48 h, repeated-measures ANOVA, W-: n = 14, W+: n = 15, *P* = 3×10^−6^), and 72 h post-cell seeding (72 h, repeated-measures ANOVA, W-: n = 14, W+: n = 14, *P* = 9.2622×10^−7^). The red and blue circles indicate the fluorescence intensity of aae-miR-34-3p per cell in W- and W+ cells, respectively. (C) Comparison of nuclear aae-miR-34-3p (two-sided t test, W-: n = 3, W+: n = 3, *P* = 0.0092) and cytoplasmic aae-miR-34-3p (two-sided t test, W-: n = 3, W+: n = 3, *P* = 0.0353) levels between W- and W+ cells by qPCR. (D) Coexpression analysis of aae-miR-34-3p (two-sided t test, W-: n = 3, W+: n = 3, *P* = 0.0004) and the *MyD88* gene (two-sided t test, W-: n = 3, W+: n = 3, *P* = 0.0151) in *Ae*. *aegypti* female mosquitoes. (E) Expression differences of aae-miR-34-3p (two-sided t test, W-: n = 3, W+: n = 3, *P* = 0.0002) and the *MyD88* gene (two-sided t test, W-: n = 3, W+: n = 3, *P* = 0.0003) in *Ae*. *aegypti* cells. (F) The representative gel picture of the western blot assay demonstrated an overexpression of *MyD88* protein in W+ cells in comparison to W- cells. β-actin protein was used as the housekeeping protein. (G) Differential expression of *MyD88* protein between W+ and W- cells (two-sided t test, W-: n = 3, W+: n = 3, *P* = 0.0231). The fold change (W+ vs. W- cells) was determined in each individual test. (H) Expression difference of the *MyD88* gene upon the upregulation of aae-miR-34-3p in W- cells at day 3 post-transfection (3D, two-sided t test, AC: agomir control treatment group, n = 5, A-34-3p: aae-miR-34-3p agomir treatment group, n = 5, *P* = 0.2×10^−5^) and day 5 post-transfection (5D, two-sided t test, AC: n = 5, A-34-3p: n = 5, *P* = 0.14×10^−4^). (I) The representative gel photo of *MyD88* protein from the AC and A-34-3p groups of W- cells in the western blot assay. (J) The relative expression of MyD88 protein in A-34-3p group compared to AC group (two-sided t test, AC: n = 4, A-34-3p: n = 4, *P* = 0.0052). (K) Fold changes in the expression of the *MyD88* gene in response to silencing of aae-miR-34-3p in W+ cells at 3 days post-transfection (3D, two-sided t test, ATC: Antagomir control treated group, n = 4, AT-34-3p: aae-miR-34-3p antagomir treated group, n = 4, *P* = 0.0162) and 5 days post-transfection (5D, two-sided t test, ATC: n = 5, AT-34-3p: n = 5, *P* = 0.0103). (L) The representative gel image of *MyD88* protein expression difference in W+ cells between AT-34-3p and ATC groups in the western blot assay. (M) The corresponding quantification of western blot images for the MyD88 protein in the AT-34-3p group related to the ATC group (two-sided t test, ATC: n = 4, AT-34-3p: n = 4, *P* = 0.0010). (N) Relative expression levels of the *MyD88* gene in aae-miR-34-3p agomir- and agomir control-treated cells at 3 days post-transfection (3D, two-way ANOVA, n = 5, W- AC vs. W+ AC: *P* = 1.0563×10^−16^, W+ A-34-3p vs. W+ AC: *P* = 1.13×10^−4^, W+ A-34-3p vs. W- AC: *P* = 1.0008×10^−18^) and 5 days post-transfection (5D, two-way ANOVA, n = 5, W- AC vs. W+ AC: *P* = 4.6183×10^−14^, W+ A-34-3p vs. W+ AC: *P* = 7.2124×10^−7^, W+ A-34-3p vs. W- AC: *P* = 1.5911×10^−17^). (O) Relative expression of the *MyD88* gene in response to the enhancement of aae-miR-34-3p expression in female mosquitoes (one-way ANOVA, n = 9, W- AC vs. W+ AC: *P* = 0.046, W+ A-34-3p vs. W+ AC: *P* = 0.0035). (N-O): W- AC indicates agomir control treated W- female mosquitoes or W- cells, W+ AC represents agomir control treated W+ female mosquitoes or W+ cells, W+ A-34-3p means aae-miR-34-3p agomir treated W+ female mosquitoes or W+ cells. The error bars indicate the standard error. F means fold change value. ****P < 0.0001; ***P < 0.001; **P < 0.01; *P < 0.05.

To better understand the aae-miR-34-3p expression regulated by *Wolbachia w*AlbB, we assayed its subcellular distribution and expression using a sequence-specific fluorescent RNA probe for RNA-FISH analysis. The fluorescence intensity of aae-miR-34-3p in W+ cells was significantly higher than that in W- cells at 24 h, 48 h, and 72 h post-cell seeding (Fig 3A and 3B). In particular, *w*AlbB led to a 2.39-fold increase at 48 h post-cell seeding (Fig 3A and 3B). Notably, aae-miR-34-3p was present in both the nucleus and cytoplasm of *Ae*. *aegypti* cells (Fig 3A). Furthermore, via nucleocytoplasmic separation of W- and W+ cells, the abundance of aae-miR-34-3p was measured separately in the nucleus and cytoplasm. The results showed that *w*AlbB increased both the abundance of nuclear and cytoplasmic aae-miR-34-3p in *Ae*. *aegypti* cells (Fig 3C).

To explore the association of aae-miR-34-3p and *MyD88*, the binding relationship between aae-miR-34-3p and the mRNA of the *MyD88* gene was demonstrated using the dual-luciferase reporter assay (S3 Fig). Subsequently, we examined the coexpression of aae-miR-34-3p and the *MyD88* gene in W+ and W- *Ae*. *aegypti* mosquitoes at day 7 post-eclosion (Fig 3D) and cell mixtures collected at day 1 to day 5 post-seeding (Fig 3E). The results showed that the *MyD88* gene and aae-miR-34-3p were coexpressed in W+ and W- *Ae*. *aegypti*. Interestingly, the mRNA levels of the *MyD88* gene were induced by *w*AlbB when aae-miR-34-3p expression was enhanced in W+ mosquitoes and cells (Fig 3D and 3E). Moreover, the protein level of *MyD88* was elevated by *w*AlbB in W+ cells (Fig 3F and 3G), implying a potential involvement of aae-miR-34-3p in the regulation of *MyD88* expression.

To evaluate the impact of aae-miR-34-3p on the expression of *MyD88*, we upregulated aae-miR-34-3p in W- cells, which have lower *MyD88* gene expression levels than W+ cells (Fig 3E). Using a synthetic aae-miR-34-3p sequence-specific agomir to enrich the abundance of aae-miR-34-3p in the cells, *MyD88* expression at the transcript level was significantly increased by 3.25-fold and 2.76-fold at day 3 and day 5 post-transfection, respectively (Fig 3H). Furthermore, *MyD88* expression at protein level was also increased by 2.14-fold at day 5 post-transfection, compared to the cells treated with a random miRNA sequence without any host target (Fig 3I and 3J). In turn, depletion of aae-miR-34-3p assay was conducted in W+ cells transfected with a synthetic antagomir with reverse-complementary sequences to aae-miR-34-3p in comparison with the cells transfected with an antagomir control. At day 3 post-transfection, significant suppression of *MyD88* at transcript level was observed in the aae-miR-34-3p antagomir-treated cells (Fig 3K). At day 5 post-transfection, obviously reduction of *MyD88* at protein level was presented in the aae-miR-34-3p antagomir-treated cells (Fig 3L and 3M). These results indicate that aae-miR-34-3p positively regulates the expression of *MyD88* at transcript and protein level in *Ae*. *aegypti* cells, supporting that *w*AlbB induces aae-miR-34-3p levels to enhance the expression of *MyD88* for activation of the Toll pathway.

To assess whether aae-miR-34-3p could further enhance *MyD88* gene expression in *Wolbachia*-infected *Ae*. *aegypti*, we conducted experiments in both cells and mosquitoes. At both day 3 and day 5 post-transfection with the agomir control, *MyD88* expression was induced by *Wolbachia* in W+ cells and W+ female mosquitoes, compared to W- cells and W- female mosquitoes, respectively (Fig 3N and 3O). Furthermore, *MyD88* expression was further enhanced in W+ cells transfected with the aae-miR-34-3p agomir, compared to W+ cells treated with the agomir control (Fig 3N). Notably, on day 5 post-aae-miR-34-3p agomir injection, a remarkable 2.08-fold increase in *MyD88* gene expression was observed in W+ female mosquitoes compared to W+ female mosquitoes with injection of agomir control (Fig 3O). These findings demonstrate that aae-miR-34-3p can augment *MyD88* gene expression in both W+ cells and mosquitoes, even when *MyD88* gene expression has already been induced by *Wolbachia*.

### *Wolbachia* suppress aae-lnc-2268 to increase the expression of aae-miR-34-3p and the *MyD88* gene

**Fig 4:**
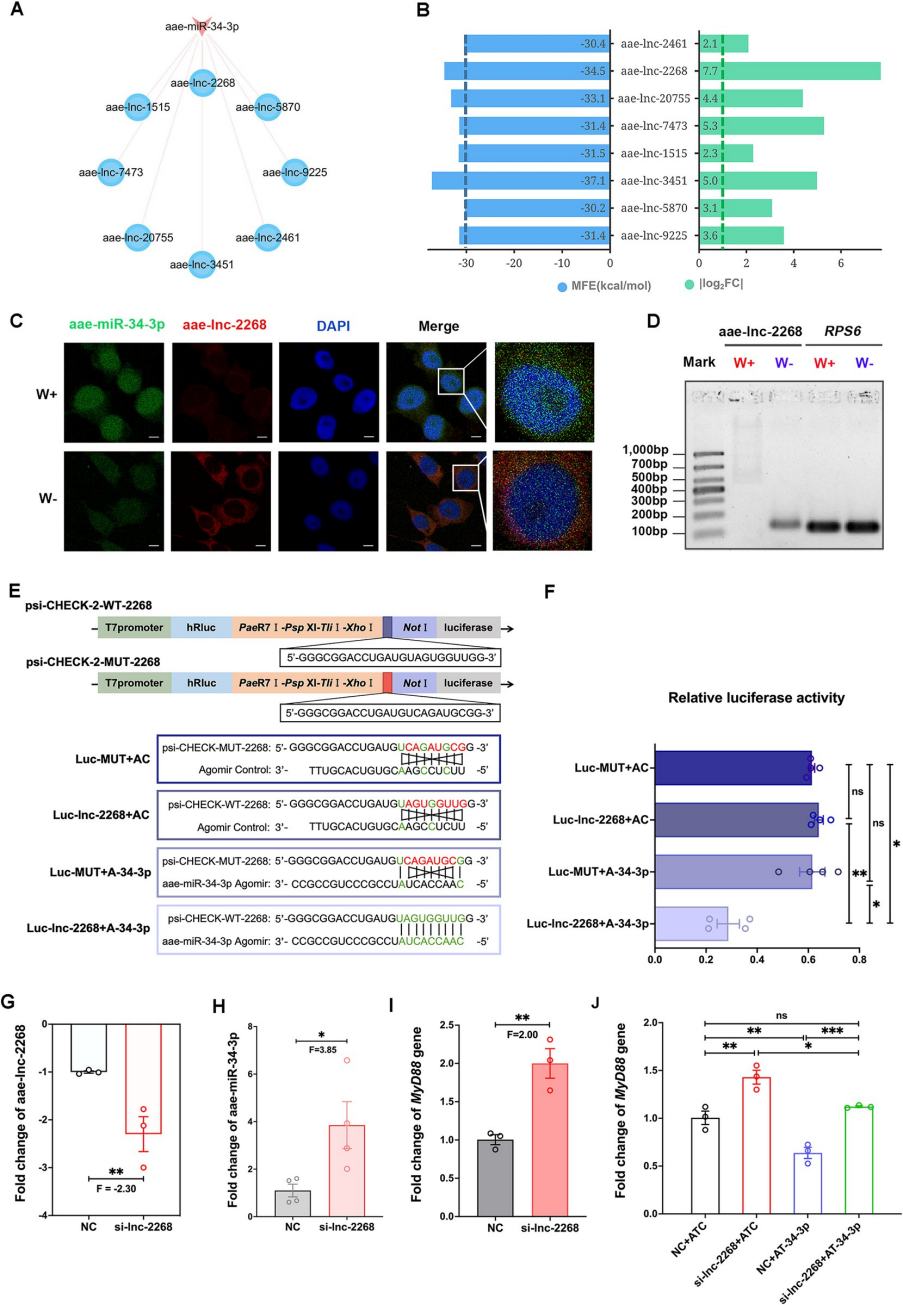
*Wolbachia w*AlbB reduces aae-lnc-2268 expression to enhance aae-miR-34-3p expression in *Ae*. *aegypti*. (A) The predicted network based on the DE lncRNAs and aae-miR-34-3p. Blue circles represent lncRNAs, and arrows indicate miRNAs. (B) The MFE value and absolute log_2_(FC) value for lncRNAs with a predicted interaction with aae-miR-34-3p. (C) Representative image of aae-miR-34-3p and aae-lnc-2268 subcellular distribution in W- and W+ cells at 63× magnification. The scale bar indicates 5 μm. (D) Image of aae-lnc-2268 presence in W- and W+ cells determined using electrophoresis. (E) Schematic representation of the construct psi-CHECK-2 plasmids used in the dual luciferase reporter assay shown in the on top panel. A schematic diagram of the predicted binding sites between aae-miR-34-3p agomir and aae-lnc-2268 plasmids in the dual luciferase reporter assay shown in the bottom panel. (F) Detection of the binding relationship between aae-lnc-2268 and aae-miR-34-3p via dual-luciferase reporter assay (one-way ANOVA, Luc-MUT+AC: n = 4, Luc-lnc-2268+AC: n = 4, Luc-MUT+A-34-3p: n = 4, Luc-lnc-2268+A-34-3p: n = 4, Luc-MUT+AC vs. Luc-lnc-2268+A-34-3p: *P* = 0.0114, Luc-MUT+AC vs. Luc-lnc-2268+AC: *P* = 0.5934, Luc-MUT+AC vs. Luc-MUT+A-34-3p: *P* = 0.9999, Luc-lnc-2268+AC vs. Luc-lnc-2268+A-34-3p:*P* = 0.0061, Luc-MUT+A-34-3p vs. Luc-lnc-2268+A-34-3p: *P* = 0.0103). Luc-MUT+AC: group cotransfected with psi-CHECK-2-MUT-2268 and agomir control, Luc-lnc-2268+A-34-3p: group with cotransfection of psi-CHECK-2-WT-2268 and aae-miR-34-3p agomir, Luc-lnc-2268+AC: group cotransfected with psi-CHECK-2-WT-2268 and agomir control, Luc-MUT+A-34-3p: group cotransfected with psi-CHECK-2-MUT-2268 and aae-miR-34-3p agomir. (G) The knockdown efficiency of aae-Inc-2268 from W- cells in the aae-lnc-2268 downregulation assay at 48 h post-transfection (two-sided t test, NC: n = 3, si-lnc-2268: n = 3, *P* = 0.0066). (H) Fold changes in aae-miR-34-3p from W- cells in the aae-lnc-2268 downregulation assay at 96 h post-transfection (two-sided t test, NC: n = 4, si-lnc-2268: n = 4, *P* = 0.0132). (I) *MyD88* gene expression change in the aae-lnc-2268 downregulation assay at 120 h post-transfection (two-sided t test, NC: n = 3, si-lnc-2268: n = 3, *P* = 0.0046). (G-I): NC indicates the siRNA negative control-transfected cells, si-lnc-2268 indicates the aae-lnc-2268 sequence-specific siRNA-treated cells. (J) Fold change in *MyD88* gene expression in the aae-Inc-2268 functional rescue assay (one-way ANOVA, NC+ATC: n = 3, si-lnc-2268+ATC: n = 3, NC+AT-34-3p: n = 3, si-lnc-2268+AT-34-3p: n = 3, NC+ATC vs. si-lnc-2268+ATC: *P* = 0.0045, NC+ATC vs. NC+AT-34-3p: *P* = 0.0010, NC+ATC vs. si-lnc-2268+AT-34-3p: *P* = 0.2429, si-lnc-2268+ATC vs. si-lnc-2268+AT-34-3p: *P* = 0.0298, NC+AT-34-3p vs. si-lnc-2268+AT-34-3p: *P* = 0.0002). NC+ATC: cells transfected with the siRNA control and antagomir control, si-lnc-2268+ATC: cells transfected with aae-lnc-2268 siRNA and antagomir control, NC+AT-34-3p: cells transfected with the siRNA control and aae-miR-34-3p antagomir, si-lnc-2268+AT-34-3p: cells transfected with aae-lnc-2268 siRNA and the aae-miR-34-3p antagomir. The error bars indicate the standard error. Each circle indicates a replicate per tested group. F means fold change (FC) value. ***P < 0.001; **P < 0.01; *P < 0.05; ns, non-significant.

To test the hypothesis that *Wolbachia* might utilize long non-coding RNA (lncRNA) to modulate the interaction of aae-miR-34-3p and the *MyD88* gene, candidate lncRNAs with potential targets to aae-miR-34-3p were predicted using RNAhybrid software. Initially, we found that 8 candidate lncRNAs might target aae-miR-34-3p with an MFE ≤ -30 kcal/mol (Fig 4A). Among them, aae-lnc-2268, a 3140 nt lincRNA, was chosen for further characterization due to the highest fold change in expression regulated by *w*AlbB based on the data reported previously [21] (Fig 4B). We firstly examined the subcellular distribution of aae-miR-34-3p and aae-lnc-2268 in the W- and W+ cell lines via RNA-FISH using florescent RNA probes. The high abundance of aae-lnc-2268 observed in the cytoplasm of W- cells was significantly reduced in the cytoplasm of W+ cells, reaching a level similar to that found in the nucleus (Fig 4C). Interestingly, while aae-miR-34-3p expression was induced by *Wolbachia* (Fig 3C and 3E), aae-lnc-2268 levels were dramatically decreased by *Wolbachia* to the detection threshold of conventional PCR and qPCR in W+ cells (Figs 4D and S4).

To verify the interactions between aae-lnc-2268 and aae-miR-34-3p, a double-luciferase reporter assay was conducted using an aae-miR-34-3p agomir and the psi-CHECK-2-WT-2268 plasmid. The recombinant plasmid was generated from the psi-CHECK-2 vector to express a mimic fragment of aae-lnc-2268, which based on prediction contained the binding sites between aae-lnc-2268 and aae-miR-34-3p (Fig 4E). As a negative control, the plasmid psi-CHECK-2-MUT-2268 was constructed to express a mutated fragment of aae-lnc-2268, which included a 7-base mutation within the binding sites (Fig 4E). Cotransfection was performed in human embryonic kidney-derived 293T cells. This cell line was chosen to eliminate the potential effect of endogenous miRNAs in *Ae*. *aegypti* cells. The results indicated that the luciferase activity derived from psi-CHECK-2-WT-2268 was significantly decreased in the cells cotransfected with the aae-miR-34-3p agomir compared to that in the control group. However, there was no difference in luciferase activity in the cells cotransfected with either psi-CHECK-2-MUT-2268 with agomir control or psi-CHECK-2-WT-2268 with the aae-miR-34-3p agomir (Fig 4F). These results indicate the direct interaction between aae-miR-34-3p and aae-lnc-2268.

To detect the impact of aae-lnc-2268 on aae-miR-34-3p expression, the aae-lnc-2268 downregulation assay was performed in W- cells using aae-lnc-2268 sequence-specific siRNA. When the abundance of aae-lnc-2268 was reduced by 2.30-fold at 48 h post-transfection (Fig 4G), the expression level of aae-miR-34-3p was increased by 3.85-fold at day 4 post-transfection in the treated group compared to that in the control group (Fig 4H). Moreover, the regulation of the *MyD88* gene by aae-lnc-2268 was also assessed. At day 5 post-transfection, the expression of the *MyD88* gene was elevated by 2.0-fold (Fig 4I).

To further verify that indirect manipulation of the *MyD88* gene was triggered by aae-lnc-2268 through aae-miR-34-3p, a functional rescue assay was conducted in W- cells by cotransfection of the aae-miR-34-3p antagomir or antagomir control with aae-lnc-2268 siRNA or the siRNA control. Similar to previous results, *MyD88* gene expression was suppressed in cells cotransfected with the siRNA control and aae-miR-34-3p antagomir, but *MyD88* gene expression was enhanced in cells cotransfected with aae-lnc-2268 siRNA and the antagomir control (Fig 4J). Interestingly, the reduced *MyD88* expression upon depletion of aae-miR-34-3p via the antagomir reagent was rescued to the same level as that in the control group in response to the silencing of aae-lnc-2268 via the siRNA reagent. These data demonstrated that aae-lnc-2268 promoted the expression of the *MyD88* gene through aae-miR-34-3p. Taken together, our results illustrate that *Wolbachia*-mediated downregulation of aae-lnc-2268 expression can either directly or indirectly, through up-regulation of aae-miR-34-3p, enhance *MyD88* gene expression.

### *Wolbachia*-induced aae-miR-34-3p expression activates the Toll pathway to inhibit DENV-2 replication

**Fig 5:**
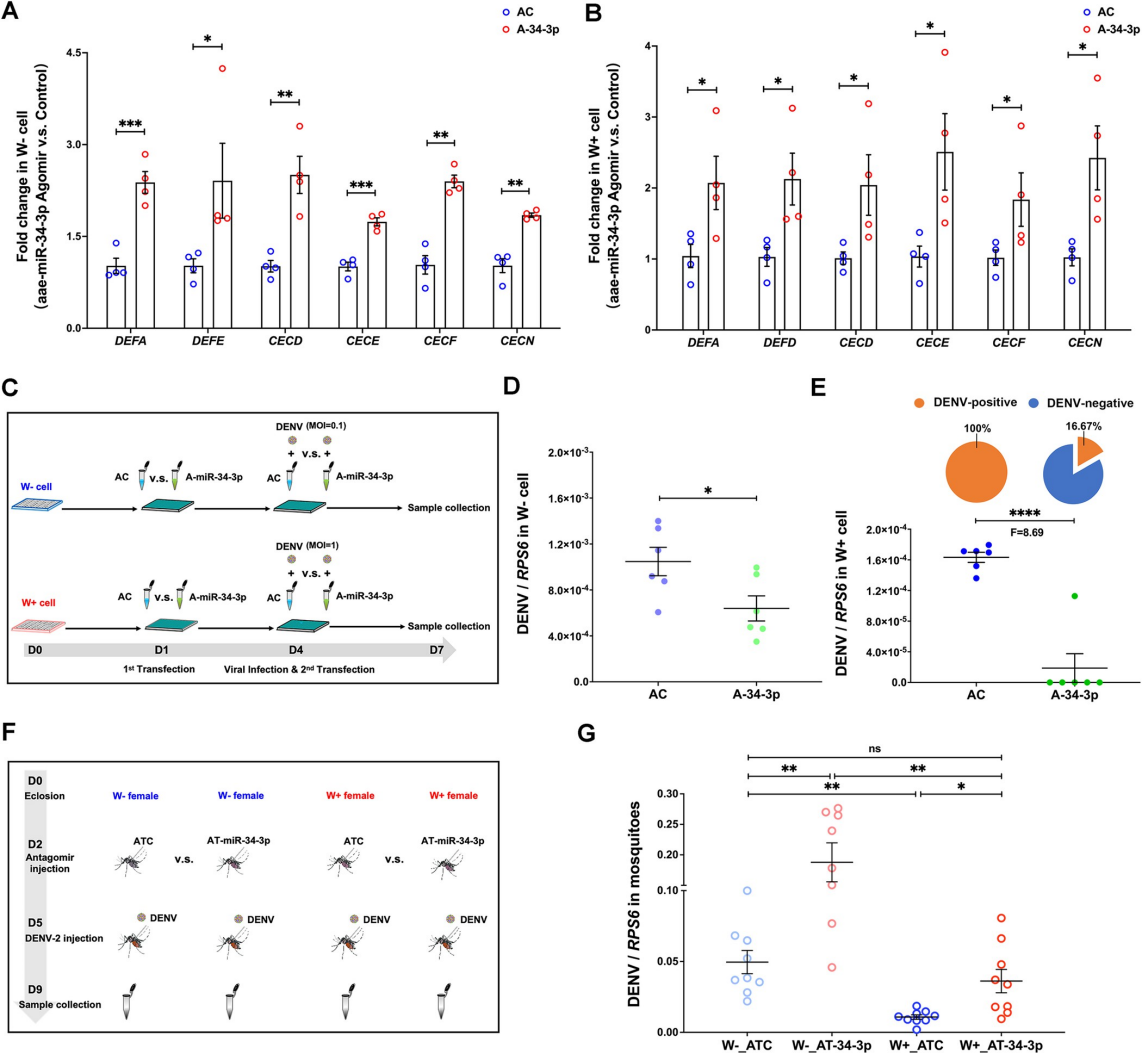
*Wolbachia w*AlbB-induced aae-miR-34-3p expression promotes antimicrobial peptide expression to inhibit DENV replication in *Ae*. *aegypti*. (A) The enhancement of *Defensin* genes (two-sided t test, AC: n = 4, A-34-3p: n = 4, *DEFA*: *P* = 0.0007, *DEFE*: *P* = 0.0173) and *Cecropin* genes (two-sided t test, AC: n = 4, A-34-3p: n = 4, *CECD*: *P* = 0.0011, *CECE*: *P* = 0.0006, *CECF*: *P* = 0.0017, *CECN*: *P* = 0.0030) in W- cells at day 5 post-transfection with aae-miR-34-3p agomir. (B) Significant increases in the expression of *Defensin* genes (two-sided t test, AC: n = 4, A-34-3p: n = 4, *DEFA*: *P* = 0.0334, *DEFD*: *P* = 0.0168) and *Cecropin* genes (two-sided t test, AC: n = 4, A-34-3p: n = 4, *CECD*: *P* = 0.0249, *CECE*: *P* = 0.0177, *CECF*: *P* = 0.0493, *CECN*: *P* = 0.0103) in W+ cells at day 5 post-transfection with aae-miR-34-3p agomir. (C) Schematic of the aae-miR-34-3p upregulation assay and viral infection assay in W- cells and W+ cells. D0, D1, D4, and D7 indicate 0, 1, 4, and 7 days post cell seeding, respectively. (D) DENV-2 infection levels in W- cells between the agomir control (AC)- and aae-miR-34-3p agomir (A-34-3p)-treated groups at 72 h post-DENV-2 infection (two-sided t test, AC: n = 6, A-34-3p: n = 6, *P* = 0.0327). (E) Differences in DENV-2 infection levels in W+ cells at 72 h post-DENV-2 infection (two-sided t test, AC: n = 6, A-34-3p: n = 6, *P* = 0.27×10^−4^). The DENV-positive infection rates are presented as pie diagram on top for each group. A-E: AC indicates agomir control treated group, A-34-3p means aae-miR-34-3p agomir treated group. F means fold change value. (F) Schematic of the aae-miR-34-3p downregulation assay and viral infection assay in mosquitoes. D0, D2, D5, and D9 indicate 0, 2, 5, and 9 days post-eclosion, respectively. (G) DENV-2 infection levels in female mosquitoes at day 7 post downregulation of aae-miR-34-3p. Compared to that of the antagomir control-injected W- (W-_ATC, n = 9) and W+ (W+_ATC, n = 9, one-way ANOVA, W-_ATC vs. W+_ATC: *P* = 0.0014) females, there was significant difference in viral infection (one-way ANOVA, W-_ATC vs. W-_AT-34-3p: *P* = 0.0031, W+_ATC vs. W+_AT-34-3p: *P* = 0.0151, W+_AT-34-3p vs. W-_AT-34-3p: *P* = 0.0018) in the aae-miR-34-3p antagomir-injected W- (W-_AT-34-3p, n = 8), and W+ female *Ae*. *aegypti* (W+_AT-34-3p, n = 9), respectively. However, there was no significant difference between the aae-miR-34-3p antagomir-injected W+ and antagomir control-injected W- *Ae*. *aegypti* (one-way ANOVA, W-_ATC vs. W+_AT-34-3p: *P* = 0.2658). The DENV-2 infection level was determined as the copy number of the DENV-2 *NS5* gene normalized to that of the *Ae*. *aegypti RPS6* gene. The line shows the mean value of the DENV infection level, and the error bar indicates the standard error. ****P < 0.0001; ***P < 0.001; **P < 0.01; *P < 0.05; ns, non-significant.

To investigate the hypothesis that *Wolbachia*-induced aae-miR-34-3p might promote the production of AMPs through activation of the Toll pathway, the effect of aae-miR-34-3p on the expression of *Cecropin* and *Defensin* family genes, the AMPs with strong antiviral activity that combat DENV through the Toll pathway induced by *Wolbachia w*AlbB [8], was measured in an *in vitro* function assay. When the aae-miR-34-3p agomir was added to W- cells to mimic the induced aae-miR-34-3p expression in W+ cells, the expression of *Defensin A* (*DEFA*), *Defensin E* (*DEFE*), *Cecropin D* (*CECD*), *Cecropin E* (*CECE*), *Cecropin F* (*CECF*), and *Cecropin N* (*CECN*) was significantly increased at day 5 post-transfection (Fig 5A).

To investigate whether the increase in AMPs, stimulated by *Wolbachia* could be further augmented by aae-miR-34-3p, we conducted a similar function assay in W+ cells. Strikingly, the expression of *DEFA*, *DEFD*, *CECD*, *CECE*, *CECF*, and *CECN* was further increased in the W+ cells treated with aae-miR-34-3p agomir (Fig 5B). This finding suggested that *Wolbachia w*AlbB increased aae-miR-34-3p expression to induce *cecropin* and *defensin* gene expression through activation of the Toll pathway, and boosting aae-miR-34-3p expression in W+ cells can further promote *Wolbachia*-mediated virus blocking in *Ae*. *aegypti*.

Subsequently, we evaluated the effect of *Wolbachia*-induced aae-miR-34-3p expression on DENV-2 infection in *Ae*. *aegypti*. In an aae-miR-34-3p function assay, the abundance of aae-miR-34-3p was initially enhanced in W- cells via transfection of aae-miR-34-3p agomir (Fig 5C). At day 3 post-transfection, the cells were subjected to a second transfection with the aae-miR-34-3p agomir or agomir control, as well as DENV-2 infection at a multiplicity of infection (MOI) = 0.1 (Fig 5C). At day 3 post-DENV-2 infection, a significant decrease in DENV genomic RNA levels was observed in the aae-miR-34-3p agomir-transfected W- cells (Fig 5D). These results indicate that over-expression of aae-miR-34-3p can result in inhibition of DENV in *Ae*. *aegypti*.

We then performed a similar assay in W+ cells to test whether *Wolbachia*-mediated DENV inhibition could be further enhanced by an increase in aae-miR-34-3p expression (Fig 5C and 5E). Notably, DENV-2 replication was suppressed by 8.69-fold in cells transfected with the aae-miR-34-3p agomir, in which the positive viral infection rate dropped to 16.67% with the complete blockade of viral infection in 5 out of 6 biological replicates at day 3 post-viral infection (Fig 5E). This suggests that the anti-DENV-2 resistance induced by *Wolbachia* can be further enhanced, and it is possible to achieve complete blocking of DENV-2 by increasing the aae-miR-34-3p level in *Ae*. *aegypti*.

To further investigate the role of aae-miR-34-3p in *Wolbachia*-induced resistance to DENV-2 in *Ae*. *aegypti* mosquitoes, we downregulated aae-miR-34-3p in female mosquitoes on day 2 post-eclosion, followed by viral infection on day 3 after treatment with antagomir (Fig 5F). As expected, *w*AlbB induced strong resistance to DENV-2, as evidence by the comparison of virus infection levels between W+ and W- females treated with the antagomir control (Fig 5G). Silencing of aae-miR-34-3p led to a significant increase in DENV-2 infection level in the group injected with aae-miR-34-3p antagomir compared to the control group in both W+ and W- mosquitoes (Fig 5G). Interestingly, aae-miR-34-3p silencing in W+ mosquitoes led to recovery of the DENV replication level to a level similar to that in antagomir control-treated W- mosquitoes, although the viral infection also increased in W- mosquito post aae-miR-34-3p silencing (Fig 5G). Taken together, these findings suggest that *Wolbachia*-induced aae-miR-34-3p plays a crucial role in *Wolbachia*-mediated anti-DENV-2 effects.

## Discussion

**Fig 6:**
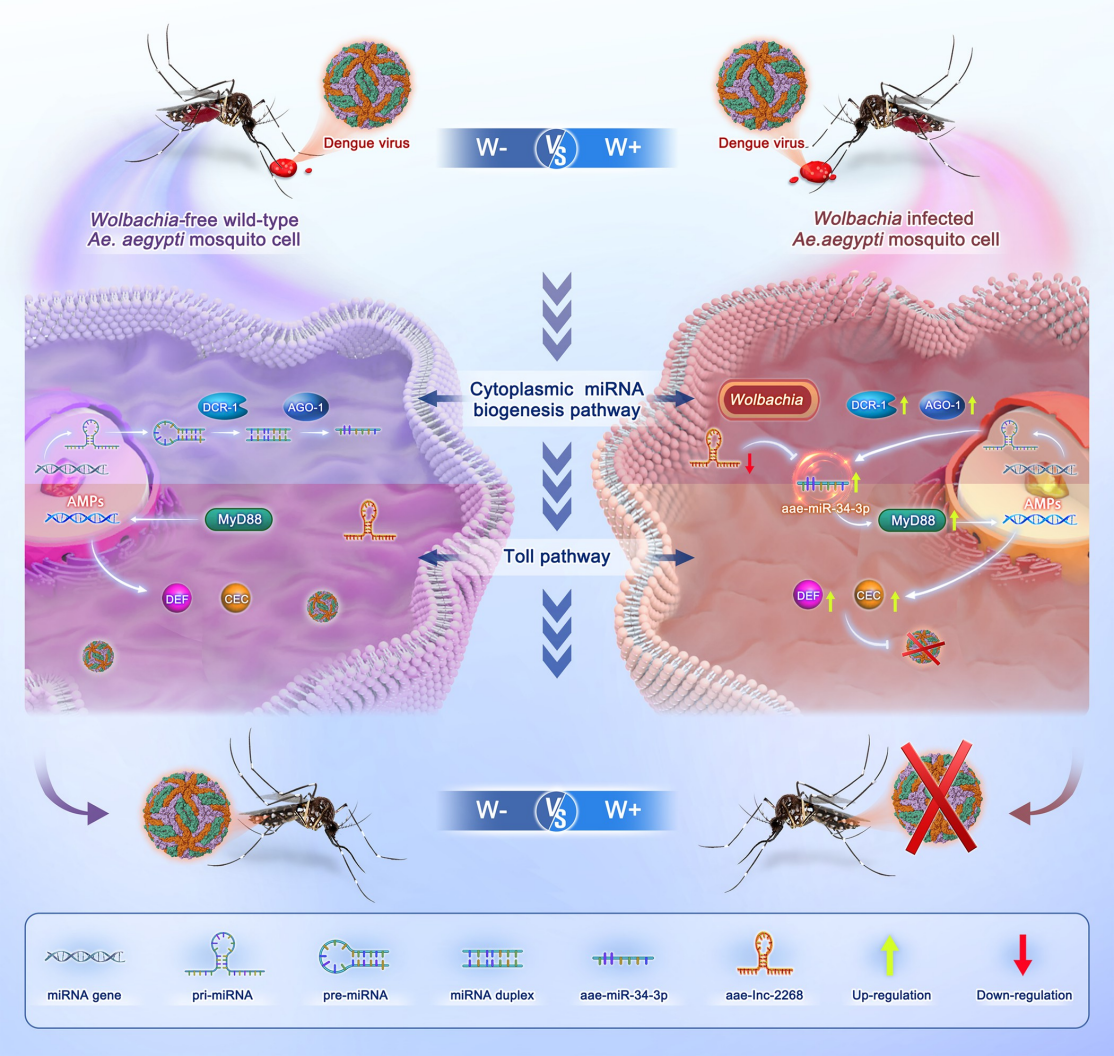
Schematic diagram of the findings in this study. The white sharp-ended arrows and the white flat-ended arrows represent the promoting effects and inhibitory effects detected in this study, respectively.

Since the symbiont *Wolbachia* has a remarkable ability to significantly inhibit or even completely block DENV transmission in its host *Ae*. *aegypti* [6,9], the mechanism underlying *Wolbachia-*mediated interference with DENV infection has garnered considerable attention. We have previously uncovered that *Wolbachia* induces ROS-dependent activation of the Toll pathway to control DENV in *Ae*. *aegypti* [8]. To extend this finding, we further investigated the hypothesis that *Wolbachia* might employ multiple antiviral mechanisms in its battle against DENV. Here, we found that *Wolbachia* induces *Dcr-1* and *Ago-1B* gene expression to modulate the cytoplasmic miRNA biogenesis pathway upon DENV-2 infection and that suppression of cytoplasmic miRNA biogenesis via depletion of *Dcr-1* strongly enhances DENV replication in female *Ae*. *aegypti*. Regarding the miRNA pathway-induced antiviral effects, we demonstrate that *Wolbachia* subgroups A (*w*MelPop) and B (*w*AlbB) increase both the nuclear and cytoplasmic levels of aae-miR-34-3p, a 23 nt immuno-microRNA in *Ae*. *aegypti*. This augmentation strengthens the Toll pathway antiviral immune response against DENV-2 by upregulating the expression of the *MyD88* gene and genes within Cecropin and Defensin families in *Ae*. *aegypti*. Moreover, we found that *Wolbachia w*AlbB suppresses aae-lnc-2268 to reduce its negative regulation of aae-miR-34-3p through direct binding, which reinforces the aae-miR-34-3p-mediated antiviral immune response, enhancing *Wolbachia*-induced antiviral effects in W+ cells. We assessed the contribution of the aae-miR-34-3p-mediated antiviral effect in female *Ae*. *aegypti* through a viral infection assay, underscoring its pivotal role in *Wolbachia*-induced interference with DENV-2 replication. Thus, we illustrate a novel mechanism by which *Wolbachia* orchestrates crosstalk between the cytoplasmic miRNA biogenesis pathway and the Toll pathway via aae-miR-34-3p, ultimately strengthening its potent antiviral effect on DENV-2 replication (Fig 6).

In the contest between *Wolbachia* and DENV, *Wolbachia* either activates Toll pathway antiviral immune responses [8,9] or employs nonimmune miRNAs dedicated to potent pathogen interference [12,17]. We have also found that *Wolbachia w*AlbB has the capacity to induce *Dcr-1* and *Ago-1* gene expression upon DENV infection in *Ae*. *aegypti*, suggesting that *Wolbachia* could manipulate miRNA maturation, stability and functionality via *Ago-1* and *Dcr-1* genes in response to DENV-2 infection. These two genes encode the key regulators of cytoplasmic miRNA biogenesis and functionality [26], which are sensitive to viral infection in insects [27,28]. During infection by Israeli acute paralysis virus (IAPV) and slow bee paralysis virus (SBPV), *Dcr-1* and *Ago-1* gene expression is increased in bees [27]. Moreover, differences in AGO-1 protein expression have been found in silkworms in response to infection with the baculovirus Bombyx mori nucleopolyhedrovirus (BmNPV) [28]. Furthermore, here, we demonstrated the essential role of the cytoplasmic miRNA biogenesis pathway via the *Dcr-1* gene in the inhibition of DENV-2 replication in W+ *Ae*. *aegypti*. Silencing the *Dcr-1* or *Ago-1* gene led to significant increases in the virus titers of DENV-2 and DENV-4, which has been previously shown in *Drosophila melanogaster* [29]. These data suggest that *Wolbachia*’s strong antiviral effect in pathogen interference is unlikely to foster resistance due to the involvement of multiple antiviral mechanisms. This reinforces the notion that *Wolbachia*-based mosquito control strategies are stable and effective in long-term field applications for mosquito control and arboviral disease prevention.

To our knowledge, this is the first work using small RNA sequencing that shows how *Wolbachia w*AlbB regulates the miRNA profile in *Ae*. *aegypti* cells. We identified 55 DE miRNAs whose expression was altered by *Wolbachia w*AlbB, supporting the finding of miRNAs modulation by *Wolbachia* in *Ae*. *aegypti* mosquito [12,30]. Among which most DE miRNAs were expressed in *Wolbachia* strain-specific patterns according to the comparison between the 55 DE miRNAs by *Wolbachia w*AlbB and DE miRNAs induced by *Wolbachia w*MelPop in *Ae*. *aegypti* cells [14]. This finding suggests that subgroup A (*w*MelPop strain) and B (*w*AlbB strain) *Wolbachia* trigger distinct alterations in the *Ae*. *aegypti* miRNA profile. In total, 2 out of the 55 DE miRNAs, namely, aae-miR-34-3p and aae-miR-1175-5p, were regulated by both *Wolbachia* strains *w*AlbB and *w*MelPop. Intriguingly, aae-miR-34-3p expression was induced in the cytoplasm by *Wolbachia w*MelPop and *w*AlbB, and it was also increased in the nucleus by *Wolbachia w*AlbB. This finding implies that nuclear-cytoplasmic shuttling allows mature aae-miR-34-3p to be imported into the nucleus from the cytoplasm. Although the potential mechanism underlying the nuclear–cytoplasmic shuttling of aae-miR-34-3p is still unclear, the broad presence of aae-miR-34-3p in the nucleus and cytoplasm enables aae-miR-34-3p to interact with mRNAs or noncoding RNAs through distinct mechanisms. Herein, we have elucidated that aae-miR-34-3p is associated with increased expression of the *MyD88* gene and negative regulation of aae-miR-34-3p via direct binding with aae-lnc-2268 in *Ae*. *aegypti*. Our findings indicate the complexity of miRNA functions in *Ae*. *aegypti*. The complex regulatory functions of miRNAs that are commonly regulated by different strains of *Wolbachia* are an interesting topic that we aim to explore in future research.

Increasing evidence has shown that nuclear miRNAs participate in two processes: transcriptional gene silencing [31] and transcriptional gene activation [12,31–33]. Our miRNA functional data provide new evidence supporting the latter function of miRNA-mediated gene activation, as aae-miR-34-3p enhanced the expression of the *MyD88* gene in *Ae*. *aegypti*. Likewise, in *Wolbachia-*host interactions, aae-miR-2940 induces host metalloprotease gene expression by binding the 3’-UTR, which benefits *Wolbachia w*MelPop growth in *Ae*. *aegypti* [11]. In fungal-plant interactions, positive gene regulation by the natural protective miRNA miR171b enables the symbiosis of *Arbuscular mycorrhizal* [34]. In virus–host interactions, miR-122 enhances hepatitis C virus replication by targeting 5′-UTR [35]. In tumorigenesis, upregulation of target genes at both mRNA and protein levels by miRNA MIR-G-1 could promote nuclear autophagy in cancer cells [36]. Although the mechanism underlying the direct interaction of aae-miR-34-3p and the *MyD88* gene is currently unknown, we speculate that nucleus-localized aae-miR-34-3p exerts its gene-activating ability through the following mechanisms: binding to transcriptional start sites, matching the sequence motif in the promoter [37,38], localizing to an enhancer region, modifying chromatin as an enhancer [32], or increase the stability of target mRNA. As the last hypothesis, *Wolbachia* reduces the sponge effect of aae-lnc-2268 on aae-miR-34-3p, resulting in increased aae-miR-34 and MyD88 in *Ae*. *aegypti*. In any case, we report that aae-miR-34-3p functions as an epigenetic modifier via transcriptional gene activation to improve the triple interaction among *Wolbachia*, *Ae*. *aegypti and* DENV.

In our previous study, we found that *Wolbachia w*AlbB utilized the competing endogenous RNA network to activate the Toll pathway in *Ae*. *aegypti* [21]. Here, we have shown the crucial role of miRNA-mediated Toll pathway activation in *Wolbachia*-induced DENV interference in *Ae*. *aegypti* cells and mosquitoes. Emerging data suggest that *Aedes* miRNAs play a role in interference with DENV replication, such as aae-miR-989 suppressing DENV replication through the regulation of *Ae*. *aegypti* atlastin (AaATL) expression [39]. In contrast to nonimmune miRNAs, aae-miR-34-3p serves as a novel enhancer to strengthen the Toll pathway antiviral immune response. Aside from enhancing the gene expression of *MyD88*, the key regulator of the Toll pathway, aae-miR-34-3p elevated the expression of *Cecropin* and *Defensin* family genes, which are important Toll pathway immune effectors with strong antiviral activity. A similar immune miRNA mechanism has been reported for aae-miR-375; however, aae-miR-375 functions as an immune inhibitor of the Toll pathway immune response that facilitates DENV-2 replication via the *Cactus* and *Rel1* genes in *Ae*. *aegypti* [31,40]. Notably, the strength of the *Wolbachia*-mediated antiviral effect could be further enhanced by increasing the abundance of aae-miR-34-3p in W+ cells, suggesting the effectiveness and feasibility of the aae-miR-34-3p-induced antiviral effect. Moreover, the relative antiviral contribution of aae-miR-34-3p could even take up 91.17% of *Wolbachia*-medicated anti-DENV effect in *Ae*. *aegypti* mosquitoes, indicating the crucial role of aae-miR-34-3p-induced antiviral effects in *Wolbachia-*mediated interference with DENV-2. It is unclear whether the aae-miR-34-3p-induced antiviral effects are pathogen-specific to DENV-2 given reports that miRNA-mediated regulation occurs in human cancer cells in a cell type-specific manner [41]. We speculate that aae-miR-34-3p-induced antiviral effects are likely not limited to DENV-2 because they strengthen the Toll pathway antiviral immune responses through multiple genes. In future research, we aim to expand our investigation to assess the impact of aae-miR-34-3p on the replication of other DENV serotypes and various mosquito-borne viral pathogens.

In conclusion, we offer a novel, safe immune miRNA with prominent antiviral capabilities that has the potential to be developed as an environmentally friendly, innovative vector control tool by reducing the susceptibility to, or transmission of, DENV in insecticide-resistant *Ae*. *aegypti* mosquitoes. As *Wolbachia*-mediated pathogen interference can be enhanced by aae-miR-34-3p, this novel small molecule is highly compatible with *Wolbachia*-based vector control strategies, which have shown their synergistic effects in strongly inhibiting or even blocking DENV replication. Further research is necessary to explore ways of enhancing the implementation of vector control strategies based on the novel miRNAs.

## Materials and methods

### Ethics Statement

All mosquito experiments and virus studies were conducted in accordance with the protocol approved by the Ethics Committee on Biomedical Research of Hunan Normal University (No. 2018–023) and Michigan State University Institutional Animal Care and Use Committees (03/14-036-00).

### Mosquitoes

The *Wolbachia* strain *w*AlbB-infected *Ae*. *aegypti* (W+ mosquito) and *Wolbachia*-free wild-type *Ae*. *aegypti* (W- mosquito) were reared at 28°C and 80% humidity with a 12-h/12-h light/dark cycle [42]. Briefly, larvae were fed a 6% (w/v) bovine liver powder solution, while adult mosquitoes were fed a 10% (w/v) sugar solution, and female mosquitoes were fed mouse blood according to standard rearing procedures [42].

### Cells

The *Wolbachia w*AlbB-carrying *Ae*. *aegypti* WAag2 cell line (W+ cell) and the Aag2 cell line (W- cell), a *Wolbachia*-free *Ae*. *aegypti* cell line, were maintained in cell culture medium (Schneider’s *Drosophila* medium with L-glutamine supplemented with 10% (v/v) fetal bovine serum (FBS) and 1% (v/v) penicillin/streptomycin) at 26°C [5,43]. In addition, the *Ae*. *albopictus* cell line C6/36 was cultured in minimal essential medium (MEM) with 10% (v/v) heat-inactivated FBS, 1% (v/v) penicillin/streptomycin, 1% (v/v) L-glutamine, and 1% (v/v) nonessential amino acids at 32°C in a 5% (v/v) CO_2_ incubator.

In addition, human embryonic kidney 293T cells were grown in Dulbecco’s modified Eagle medium (DMEM) with 10% (v/v) FBS and 1% (v/v) penicillin/streptomycin at 37°C under 5% (v/v) CO_2_, as previously described [21].

### RNA interference in mosquitoes

First, dsRNA was synthesized using a MEGAscript T7 transcription kit (Invitrogen, TX, USA) and purified using a MEGAclear kit (Invitrogen) according to the manufacturer’s instructions. T7 promoter sequences (TAATACGACTCACTATAGGG) were incorporated in both forward and reverse primers to amplify the *Ae*. *aegypti Dicer-1* gene (forward: 5′-TCCGTTATGGATCACCCACT-3′; reverse: 5′-TGTTTTTGCCGTTTGAGGAT-3′) and *GFP* (forward: 5′-GGAGAAGAACTTTTCACTGG-3′; reverse: 5′-AGTTGAACGGATCCATCTTC—3′). mRNA downregulation via RNA interference was conducted through adult mosquito thorax injection, according to the standard methodology [44]. A total of 69 nL of 4 μg/μL dsRNA of DCR or GFP was injected into the thorax of CO_2_-anesthetized 1- to 2-day-old female mosquitoes using a nanoinjector. At day 3 post-dsRNA injection, some females were dissected for midgut sample collection used in evaluation of gene knockdown efficiency and the remaining females from each group were infected with DENV-2 through oral feeding of a blood meal. At day 7 post-DENV infection, the midguts were dissected to measure the DENV infection level. There were 6 samples for each group with 3 midguts per sample.

### miRNA function assay

The aae-miR-34-3p sequence-specific agomir and antagomir were synthesized by GenePharma (http://www.genepharma.com), and the sequence information is listed in S3 Table. Agomirs are chemically modified double-stranded RNAs that can mimic mature endogenous miRNAs for use in miRNA upregulation assays. In contrast, antagomirs are chemically modified single-stranded RNAs that can silence endogenous miRNAs for use in miRNA downregulation assays.

In the *in vivo* function test, the aae-miR-34-3p agomir/antagomir was injected into the thorax of CO_2_-anesthetized female mosquitoes at day 3 post-eclosion at a dose of 800 μM in a volume of 207 nL using the same method as mentioned above. Female mosquitoes were injected with the same dose of the miRNA control reagent, which is a random sequence that cannot bind to the genome of *Ae*. *aegypti*. To test the impact of aae-miR-34-3p on mRNA expression, whole mosquito samples (one mosquito as one sample) were collected at day 5 post-injection. To explore the role of aae-miR-34-3p in DENV infection, the females from each group were subjected to DENV-2 infection via thorax injection at 3 days post-injection.

In the *in vitro* function test, the cells were seeded in 48-well and 96-well cell plates 24 h prior to transfection at a density of 2×10^5^ and 1×10^5^ cells per well, respectively. A total of 800 nM miRNA agomir/antagomir with 0.5 μL of DharmaFECT Transfection reagent (Thermo Scientific, KS, USA) in 20 μL of Schneider’s *Drosophila* medium were incubated for 20 minutes at room temperature. Subsequently, the transfection reagent-miRNA agomir/antagomir complexes were gently added into each well of cells, according to the manufacturer’s instructions. To detect the role of miRNA on Toll pathway-related gene expression, the cell samples from 96-well plates were collected at day 3 and day 5 post-transfection for RNA extraction, and cell samples from 48-well plates were collected at day 5 post-transfection for protein extraction. To analyze the effect of miRNA on DENV replication, the cells were subjected to DENV infection with a second transfection of 800 nM miRNA reagent at day 3 post-transfection.

### DENV infection

The New Guinea C (NGC) strain of DENV serum type 2 was used for *Ae*. *aegypti in vivo* and *in vitro* viral infection assays. Initially, C6/36 cell monolayers at 80% confluence were infected with a DENV-2 stock (10^7^ pfu/mL) at an MOI of 1.0 at 32°C for 1 h, and then viral culture medium (MEM supplemented with 2% heat-inactivated FBS, 1% penicillin/streptomycin, 1% L-glutamine, and 1% nonessential amino acids) was added to the cell monolayers after the removal of DENV-2. Subsequently, DENV-2 was propagated in C6/36 cells at 32°C with 5% (v/v) CO_2_ and collected at 7 days post-infection for the viral infection assay.

In the *in vivo* viral infection assay, mosquitoes were infected with DENV-2 through either oral feeding or thorax injection. For DENV-2-infected blood oral feeding, the freshly collected DENV-2 supernatant mixed with commercial defibrinated sheep blood (Colorado Serum Company, CO, USA) in a 1:1 ratio was maintained at 37°C for 30 min prior to the blood meal. The virus-containing blood meal was offered to female mosquitoes for 45 mins through glass feeders covered with a membrane of porcine intestine, and the feeders were connected to a circulating water bath (Fisher) at 37°C. Subsequently, the mosquitoes were anesthetized immediately post-feeding using CO_2_, and only the fully engorged mosquitoes were chosen and transferred into a new waterproof cardboard container for further testing. For the DENV-2 infection assay via thorax injection, thorax injection with 69 nL of DENV-2 (10^7^ PFU/mL) was performed on each female mosquito (day 3 post-injection of the miRNA antagomir reagent) using a nanoinjector (Thermo Fisher Scientific). At day 7 post-antagomir injection, whole mosquito samples (one mosquito as one sample) were collected for testing.

In the *in vitro* viral infection assay, DENV-2 infection was performed in W+ cells and W- cells at day 3 post-transfection of the miRNA agomir or antagomir reagent with an MOI = 1 and MOI = 0.1, respectively. The higher viral dose for W+ cells was because *Wolbachia* mediated a strong antiviral effect against DENV in W+ cells. The infected cell samples were collected at day 3 post-DENV infection.

### Detection of differential expression of miRNA pathway genes

The transcript profiles of the midgut and carcass (remaining tissues except the midgut) from *Wolbachia w*AlbB-infected and uninfected female *Ae*. *aegypti* at 12 days post-DENV infection were downloaded [8] and used for the detection of *Wolbachia w*AlbB-regulated miRNA pathway regulator genes upon DENV-2 infection. The differentially expressed genes were identified based on a threshold defined as the absolute value of log_2_(fold-change) ≥0.8.

To validate the differential expression of mRNA in microarray data, the viral infection assay was carried out via DENV-containing blood meal in W+ and W- female mosquitoes at 7 days post-eclosion. At 12 days post-DENV infection, 10 midguts and corresponding carcasses were dissected and pooled as one sample. There were 3 samples for each group. Sample homogenization was performed in 600 μL RLT buffer provided by the RNeasy Mini Kit (QIAGEN Sciences, MD, USA) on ice for 2 min using a disposable sterile enzyme-free pestle. The processed mosquito samples were used for subsequent RNA extraction, cDNA synthesis and quantitative real-time PCR.

### Measurement of the differential expression of mRNA via quantitative real-time PCR

Total RNA was extracted from mosquitoes or cells using an RNeasy Mini Kit (QIAGEN Sciences), and then cDNA was synthesized using a QuantiTect Reverse Transcription Kit (QIAGEN Sciences) according to the manufacturer’s recommendations [5]. Quantitative real-time PCR was conducted using a Quantities SYBR Green PCR Kit (QIAGEN Sciences) and an ABI Prism 7900HT Sequence Detection System. The primers for the ribosomal protein S6 (*RPS6*) gene [8] and *NS5* gene of DENV-2 [9] were described previously. The other primers used for qPCR are listed in S4 Table. The real copy numbers of the *NS5* and *RPS6* genes were detected based on the standard curve generated using ten-fold serial dilutions from 1×10^8^ to 1×10^1^ copies/μL of the DNA plasmid containing the *NS5* or *RPS6* gene, respectively. The *RPS6* gene was used for normalization of cDNA templates. In addition, the relative quantification of other mRNAs was carried out according to the Ct value using the 2^-ΔΔCT^ method.

### Small RNA sequencing

Total RNA was extracted from W+ and W- cells for three biological replicates of pooled cell mixtures from day 1 to day 5 post-cell passage using TRIzol reagent (Invitrogen) per the manufacturer’s protocol. The quality and quantity of RNA samples were assessed by a NanoDrop 2000 Spectrophotometer (Thermo Fisher Scientific) and a Bioanalyzer 2100 System with an RNA Pico 6000 Assay Kit (Agilent Technologies, CA, USA). Subsequently, the miRNA library and small RNA sequencing were constructed at Biomarker Technologies. In brief, 1 μg of total RNA for each sample was used in the synthesis of a small RNA library, according to the manufacturer’s recommendation for the NEBNext Small RNA Library Prep Set (New England Biolabs, USA). Specialized adaptors were used to ligate both ends of the cDNA fragments, introducing a unique barcode for each library. Library quality was evaluated using the Agilent Bioanalyzer 2100 system. The validated libraries were sequenced on the Illumina HiSeq2500 at Biomarker Technologies, resulting in 50 bp single-end reads. No spike-in controls were used in the construction of this dataset.

### miRNA quantification and differential expression analysis

The raw sequencing data underwent quality assessment to generate clean reads by filtering out adapter-containing reads, poly-N-containing reads, and low-quality reads. Subsequently, the high-quality 18- to 30-nt clean reads were filtered for elimination of ribosomal RNA (rRNA), transfer RNA (tRNA), small nuclear RNA (snRNA), small nucleolar RNA (snoRNA), other ncRNA, and repetitive sequences by Bowtie software (http://bowtieapp.com/) based on the SILVA ribosomal RNA database (https://www.arb-silva.de), Genomic tRNA Database (GtRNAdb), Rfam database (http://www.sanger.ac.uk/Software/Rfam), and Repbase (https://www.girinst.org/repbase). The remaining clean reads that could be mapped to *Ae*. *aegypti* reference genome (GCF_002204515.2) from NCBI (https://www.ncbi.nlm.nih.gov/) and matched to known *Ae*. *aegypti* miRNAs from miRbase (http://www.mirbase.org) were used for further analysis.

The transcripts per million (TPM) normalization method [45,46] was employed for the determination of miRNA expression levels. Then, the DE miRNAs were defined based on |log_2_(fold-change)| ≥1 and false discovery rate(FDR)≤0.05 by DESeq2 software [47]. Moreover, a heat map of top 15 up-regulated and down-regulated miRNAs was generated based on the TPM value in each sample using TBtools software (https://github.com/CJ-Chen/TBtools/releases) [48].

### Validation of differentially expressed miRNAs via real-time quantitative PCR

The miRNAs were extracted from female mosquitoes (day 7 post-eclosion without blood feeding) and cell samples (pooled samples with cells collected from day 1 to day 5 post-cell passage) using a miPure Cell/Tissue miRNA Kit (Vazyme, Nanjing, China) according to the manufacturer’s protocol. Following treatment with DNase I (Invitrogen), the purified miRNAs were converted to cDNA using a thermal cycler (Bio-Rad, CA, USA) with a Mir-X miRNA First-Strand Synthesis Kit (Takara, Kusatsu, Japan). The DE miRNAs were verified with the CFX96 PCR detection system (Bio-Rad) using TB Green Advantage qPCR Premix (Takara). The miRNA sequence-specific forward primers are summarized in S5 Table, and the universal reverse primer from the Mir-X miRNA First-Strand Synthesis Kit (Takara) is complementary to the 3’-end universal tag sequence of miRNAs. The thermocycling conditions were as follows: 95°C for 30 sec of denaturation, followed by 40 cycles at 95°C for 5 sec and 60°C for 30 sec. Finally, the relative quantification of miRNA was carried out according to the Ct value using the 2^-ΔΔCT^ method with the *S7* and *S5* gene serving as the reference gene for mosquito and cell samples, respectively. In addition, the expression of miRNAs was normalized to the amount of cDNA template (100 ng), and the results are shown in the supporting information.

### Detection of nuclear and cytoplasmic miRNA

The cell samples were collected using 200 μL of ice-cold lysis buffer from a Cytoplasmic and Nuclear RNA Purification Kit (Norgen BioTek, ON, Canada) with three replicates. Nuclear and cytoplasmic RNA fractions from cell samples were isolated according to the manufacturer’s instructions. The effectiveness of cellular separation was ensured by assessment of the cytoplasmic and nuclear markers actin and U6, respectively. Subsequently, cDNA synthesis and amplification were conducted as described above via qPCR, and the relative quantification of cytoplasmic and nuclear miRNA was performed using the 2^-ΔΔCT^ method with the reference gene of *actin* and *U6* gene, respectively.

### Western blot analysis

Proteins were extracted from W+ and W- *Ae*. *aegypti* cells at day 5 post-transfection or day 5 post-passage. For each group, 2×10^6^ cells per sample were collected in 200μL lysis buffer containing 1% (v/v) halt proteinase inhibitor cocktail, 0.1% (v/v) phosphatase inhibitor, and 0.5% (v/v) 100 mM PMSF from the whole protein extraction kit (KeyGEN BioTECH, Jiangsu, China) according to the manufacturer’s protocol recommendation. Following a 30-min incubation on ice, the lysed cells were centrifuged at 12,000 × g for 10 min at 4°C and the supernatant was collected. Protein was then quantified using the BCA protein assay kit (KeyGEN BioTECH).

After incubation at 100°C for 10 min with the protein loading buffer (Coolaber, Beijing, China), the weighted proteins were subjected to 10% SDS-PAGE gel electrophoresis at a constant voltage of 120 V, and then transferred to PVDF membranes (Millipore, MA, USA) at a constant 300 mA. The membranes were blocked with 5% non-fat milk for 2 h and then incubated overnight at 4°C with primary antibodies, including the mouse monoclonal anti-MyD88 antibody (Proteintech, Wuhan, China, 67969-1-Ig, 1:500) and the mouse monoclonal anti-β-actin antibody (Proteintech, 67969-1-Ig, 1:1000). After being washed three times for 10 min each with TBS-T (0.1% Tween-20) at room temperature, the membranes were incubated with horseradish peroxidase (HRP)-labeled goat anti-mouse IgG secondary antibody (Abbkine, China, 1:5000) at room temperature for 2 h. The ECL staining fluid (Abbkine) was used for imaging on the ChemiDoc MP Imaging System (BIO-RAD). The relative protein expression was compared between the W+ and W- cell groups, agomir and agomir control-treated groups, antagomir and antagomir control groups, according to the gray values of the protein bands measured by ImageJ software. Normalization was performed using the loading control of β-actin.

### Prediction of binding sites for RNA interaction

RNAhybrid [24] and RNA22 [25] software were used to search for potential binding sites between Toll pathway regulator gene mRNA and 2 DE miRNAs in *Ae*. *aegypti* genome, based on the three main criteria, namely, a complementarity of seed region ≥ 7, an MFE < -25 kcal/mol and *P* < 0.05. Moreover, the possible interaction between lncRNA and aae-miR-34-3p was predicted according to the threshold MFE value ≤ -30 kcal/mol, *P* < 0.05 and |log_2_(fold-change)| ≥1, where fold change refers to the fold change of lncRNA expression level between W+ and W- cells.

### Measurement of lncRNA expression via quantitative PCR

lncRNA was isolated from mixed cell samples from day 1 to day 5 post-cell passage via a miPure Cell/Tissue miRNA Kit (Vazyme) and then used for cDNA synthesis with a PrimeScript RT Reagent Kit (Takara). lncRNA quantification was conducted on a CFX96 PCR detection system (Bio-Rad) using TB Green Advantage qPCR Premix (Takara) with the sequence-specific primers listed in S5 Table. The qPCR conditions were as follows: initial denaturation at 95°C for 3 min, followed by 40 amplification cycles of denaturation at 95°C for 10 sec, 55°C for 30 sec of annealing, and 72°C for 30 sec of extension. The relative expression of lncRNAs was analyzed using the 2^-ΔΔCT^ method, with the *RPS6* gene serving as the reference gene.

### Detection of lncRNA via conventional PCR and electrophoresis

The expression of lncRNA was detected by conventional PCR using GoTaq Green Master Mix (Promega, Madison, WI, USA) with the primers listed in S5 Table. The PCR conditions were set as follows: initial denaturation at 95°C for 2 min, followed by 35 cycles of 95°C for 45 s, 60°C for 30 s, and 72°C for 15 s, extension at 72°C for 5 min, and 4°C until collection. The *RPS6* gene was used to confirm the quality of the cDNA samples. The PCR products were evaluated via 2% agarose gel electrophoresis with GelRed (Vazyme) staining. The gel image of PCR products was visualized and analyzed using Gel Doc XR+ (Bio-Rad).

### Fluorescence in situ hybridization (FISH)

The cell samples from each treatment group with 4 biological replicates were fixed in 8-well cell culture chamber slides (Biologix, China) with 4% paraformaldehyde (Solarbio, Beijing, China) at 4°C for 1 h and then washed 3 times with PBST [0.1% (v/v) Tween 20 in PBS]. Permeabilization was performed using 100 μL of 0.1% Triton X-100 (GenePharma, Suzhou, China) at room temperature for 15 min. To visualize the distribution of *Wolbachia* and aae-miR-34-3p, hybridization was conducted with 1 μM 6-carboxyfluorescein (FAM)-labeled aae-miR-34-3p probe and 1 μM Sulfo-Cyanine3 (Cy3)-labeled 16S rDNA *Wolbachia* probe [7] (synthesized by GenePharma) in buffer E (RNA FISH Kit, GenePharma) at 37°C for 24 h, following the manufacturer’s instructions. In addition, to visualize the intracellular localization of aae-lnc-2268 and aae-miR-34-3p, hybridization was performed with 5 μM Cy3-labeled aae-lnc-2268 probe (synthesized by GenePharma) and 1 μM FAM-labeled aae-miR-34-3p probe in buffer E at 37°C for 48 h in the dark. After washing, the cells were stained with 100 μL of 4’,6-diamidino-2-phenylindole (DAPI, 1 μg/mL, included in the kit) at room temperature for 15 min in the dark. The samples were viewed on a Leica Confocal Microscope system (Leica TCS SP8, Wetzlar, Germany). The Leica Application Suite software was used to capture 3–4 images randomly for each sample from different groups. The images were taken with the same laser intensity value, laser power, master gain value, digital offset value, and digital gain value under each fluorescence channel to ensure consistency for comparison. Fluorescence intensity was quantified using ImageJ software [49] and normalized to cell number, based on 14 individual samples per group. Probe sequences are summarized in S6 Table.

### lncRNA functional assay

A lncRNA functional assay was performed when the monolayer cells reached 80% confluence in 96-well plates. In the lncRNA downregulation assay, aae-lnc-2268 sequence-specific siRNAs or negative controls (synthesized by GenePharma Co., Ltd. S3 Table) at 300 nM per well were transfected with Lipofectamine 2000 reagent following the manufacturer’s instructions. The cell samples at 48 h, 96 h, and 120 h post-transfection were used for the measurement of lncRNA, miRNA, and mRNA expression, respectively.

In the functional rescue assay, cells were first transfected with aae-lnc-2268 siRNAs or the negative control using Lipofectamine 2000 reagent (Invitrogen). At 48 h post-transfection, the cells were subjected to a second transfection with 800 nM aae-miR-34-3p antagomir or negative control antagomir. Four groups of transfected cells were used for comparison: aae-lnc-2268 siRNA with the aae-miR-34-3p antagomir, aae-lnc-2268 siRNA with the negative control antagomir, negative control siRNA with the aae-miR-34-3p antagomir, and negative control siRNA with the negative control antagomir. At 72 h after the second transfection, cell samples were collected for further analysis.

### Dual-luciferase reporter assay

In the investigation of the binding relationship between the *MyD88* gene and aae-miR-34-3p, psi-CHECK-2-WT-MyD88 and psi-CHECK-2-MUT-MyD88 plasmids were employed to express a 25-nt predicted binding sequence and a 24-nt binding sequence with 8 base mutations, respectively. In addition, the psi-CHECK-2-WT-2268 and psi-CHECK-2-MUT-2268 plasmids were constructed to express a 24-nt predicted binding sequence and a mutated binding sequence (7 base mutations) between the aae-lnc-2268 and aae-miR-34-3p, respectively. The above luciferase reporter plasmids were constructed by Sangon Biotech (Shanghai, China). The predicted or mutated binding site sequences were cloned into the *Not*I and *Xho*I restriction sites located downstream of the *Renilla* luciferase translational stop codon in the 3’-UTR of the psi-CHECK-2 vector.

293T cells in 24-well plates were cotransfected with 500 ng of plasmid and 140 nM miRNA agomir (aae-miR-34-3p agomir or the negative control agomir) using Lipofectamine 2000 reagent (Invitrogen). The relative luciferase activities were detected using a dual−luciferase reporter assay system (Promega) at 48 h post-transfection and normalized to the internal reference of firefly luciferase activity.

### Quantification and statistical analysis

Repeated measures ANOVA was used to determine the difference in miRNA intensity between W+ and W- cells at three distinct time points. Two-way ANOVA was utilized to assess the alteration of MyD88 expression between W+ and W- cells in response to the different treatment in the miRNA upregulation assay. One-way ANOVA was used for pairwise comparisons among multiple groups in comparisons of the relative luciferase activity in the dual−luciferase reporter assay, lncRNA rescue assay, and DENV infection assay. Student’s t test was used for pairwise comparisons between two groups of mRNAs, miRNAs and lncRNAs, and the differences in DENV infection levels between two groups in *Ae*. *aegypti*. A *P* value lower than 0.05 was considered to indicate statistical significance. The experimental results are expressed as the mean ± standard error of the mean (mean ± SEM). All data were analyzed with GraphPad Prism 9.0 and IBM SPSS statistic 25.0 software.

## Supporting information

S1 Table Small RNA sequencing information. (DOCX)

S2 Table The differentially expressed miRNAs in *Ae*. *aegypti* induced by *Wolbachia w*AlbB. (DOCX)

S3 Table The sequence-specific reagents used in the miRNA and lncRNA function assays. (DOCX)

S4 Table The sequence of mRNA primers used in qPCR. (DOCX)

S5 Table The primer sequence used in quantification of noncoding RNAs via PCR. (DOCX)

S6 Table The fluorescent RNA probes used in FISH. (DOCX)

S1 Fig Induced miRNAs identified by RNA sequencing analysis of both cytoplasm and whole cell using qPCR with normalization of weighted cDNA templates. The expression of 6 out of the top 15 up-regulated DE miRNAs was increased in whole-cell samples (left panel, two-sided t test, W-: n = 4, W+: n = 4, aae-miR-989: *P* = 0.0005, aae-miR-980-5p: *P* = 0.0411, aae-miR-980-3p: *P* = 0.0156, aae-miR-34-3p: *P* = 0.0092, aae-miR-277-3p: *P* = 8.4345×10^−5^, aae-miR-2765: *P* = 0.0025) and cytoplasm samples (right panel, two-sided t test, W-: n = 3, W+: n = 3, aae-miR-989: *P* = 0.0005, aae-miR-980-5p: *P* = 0.0043, aae-miR-980-3p: *P* = 0.0002, aae-miR-34-3p: *P* = 0.0026, aae-miR-277-3p: *P* = 1.3807×10^−6^, aae-miR-2765: *P* = 0.0002) from W+ cells in qPCR analysis. The expression of miRNAs was normalized to the amount of cDNA template (100 ng). The error bars indicate the standard error. ****P < 0.0001; ***P < 0.001; **P < 0.01; *P < 0.05.
(TIF)

S2 Fig Suppressed miRNAs identified by RNA sequencing analysis both cytoplasm and whole cell using qPCR with normalization of weighted cDNA templates. qPCR analysis showed that the expression of 6 out of the top 15 downregulated DE miRNAs was decreased in whole-cell samples (left panel, two-sided t test, W-: n = 4, W+: n = 4, aae-miR-9a: *P* = 0.0043, aae-miR-281-5p: *P* = 0.0010, aae-miR-252-5p: *P* = 0.0449, aae-miR-2a-3p: *P* = 0.0192, aae-miR-12-5p: *P* = 0.0089, aae-miR-1175-5p: *P* = 2.1435×10^−6^) and cytoplasm samples (right panel, two-sided t test, W-: n = 3, W+: n = 3, aae-miR-9a: *P* = 0.0130, aae-miR-281-5p: *P* = 0.0005, aae-miR-252-5p: *P* = 0.0087, aae-miR-2a-3p: *P* = 0.0058, aae-miR-12-5p: *P* = 0.0002, aae-miR-1175-5p: *P* = 0.0033) from W+ cells in small RNA sequencing. The expression of miRNAs was normalized to the amount of cDNA template (100 ng). The error bars indicate the standard error. ****P < 0.0001; ***P < 0.001; **P < 0.01; *P < 0.05.
(TIF)

S3 Fig The binding relationship between aae-miR-34-3p and *MyD88* gene. (A) Schematic representation of the construct psi-CHECK-2 plasmids used in the dual luciferase reporter assay shown in the upper panel. A schematic diagram of the predicted binding sites between aae-miR-34-3p agomir and *MyD88* plasmids in the dual luciferase reporter assay shown in the bottom panel. (B) The binding relationship between *MyD88* and aae-miR-34-3p was determined via a dual-luciferase reporter assay (one-way ANOVA, Luc-MUT+AC: n = 6, Luc-MyD88+AC: n = 6, Luc-MUT+A-34-3p: n = 6, Luc-MyD88+A-34-3p: n = 6, Luc-MUT+AC vs. Luc-MyD88+A-34-3p: *P* = 0.0326, Luc-MUT+AC vs. Luc-MyD88+AC: *P* = 0.8503, Luc-MUT+AC vs. Luc-MUT+A-34-3p: *P* = 0.9680, Luc-MyD88+AC vs. Luc-MyD88+A-34-3p:*P* = 0.0055, Luc-MUT+A-34-3p vs. Luc-MyD88+A-34-3p: *P* = 0.0363). Luc-MUT+AC: group cotransfected with psi-CHECK-2-MUT-MyD88 and agomir control, Luc-MyD88+A-34-3p: group with cotransfection of psi-CHECK-2-WT-MyD88 and aae-miR-34-3p agomir, Luc-MyD88+AC: group cotransfected with psi-CHECK-2-WT-MyD88 and agomir control, Luc-MUT+A-34-3p: group cotransfected with psi-CHECK-2-MUT-MyD88 and aae-miR-34-3p agomir. The error bars indicate the standard error. Each circle indicates a replicate per tested group. **P < 0.01; *P < 0.05; ns, non-significant.
(TIF)

S4 Fig The Ct value of aae-lnc-2268 in W- and W+ cells determined by qPCR. The black line indicates the mean Ct value. Each circle indicates a Ct value for aae-lnc-2268 or the *RPS6* gene. The red dashed line indicates the threshold Ct value of 40.
(TIF)

S1 Raw Images Raw images in this study. (PDF)

S1 Data Supporting data underlying the figures. (XLSX)
