# Supplementary material for: Wolbachia mediates crosstalk between miRNA and Toll pathways to enhance resistance to dengue virus in Aedes aegypti
Source: PLoS Pathog. 2024 Jun 17;20(6):e1012296. doi: 10.1371/journal.ppat.1012296 (PMC11213346; doi:10.1371/journal.ppat.1012296)
Supplement: S1 Table — (DOCX) [file ppat.1012296.s001.docx]

**S1 Table.** **Small RNA sequencing information.**

| **Samples** | **Clean Reads** | **ncRNA** |
| --- | --- | --- |
| W+ S1 | 9,903,835 | 288,072(2.91%) |
| W+ S2 | 10,206,419 | 325,126(3.19%) |
| W+ S3 | 10,286,774 | 394,984(3.84%) |
| W- S1 | 10,555,245 | 525,558(4.98%) |
| W- S2 | 14,508,401 | 733,284(5.05%) |
| W- S3 | 12,357,838 | 659,063(5.33%) |

Note: ncRNA: including ribosomal RNA (rRNA), transport RNA (tRNA), small nuclear RNA (snRNA), small nucleolar RNA (snoRNA) and other ncRNAs.
