# Supplementary material for: Wolbachia mediates crosstalk between miRNA and Toll pathways to enhance resistance to dengue virus in Aedes aegypti
Source: PLoS Pathog. 2024 Jun 17;20(6):e1012296. doi: 10.1371/journal.ppat.1012296 (PMC11213346; doi:10.1371/journal.ppat.1012296)
Supplement: S2 Table — (DOCX) [file ppat.1012296.s002.docx]

**S2 Table.** **The differentially expressed miRNAs in *Ae. aegypti* induced by *Wolbachia w*AlbB.**

| **#ID** | **FDR** | **log2FC** | **Regulation** |
| --- | --- | --- | --- |
| aae-miR-184 | 0.0000029 | 1.206298464 | up |
| aae-miR-283 | 1.41E-44 | -1.668716208 | down |
| aae-miR-92b-3p | 6.4E-13 | 1.249865112 | up |
| aae-miR-13-3p | 1.13E-12 | -1.331653659 | down |
| aae-miR-190 | 9.21E-30 | 1.586124639 | up |
| aae-miR-34-5p | 6.02E-22 | 1.300450688 | up |
| aae-miR-92a-3p | 2.22E-08 | 1.25035912 | up |
| aae-miR-87 | 8.07E-43 | 2.296552554 | up |
| aae-miR-989 | 1.05E-19 | 2.359186642 | up |
| aae-miR-71-3p | 7.29E-09 | -1.219366718 | down |
| aae-miR-999 | 6.35E-127 | 6.427023707 | up |
| aae-miR-277-3p | 2.51E-102 | 3.052308556 | up |
| aae-miR-2a-3p | 2.45E-84 | -2.427525856 | down |
| aae-miR-137 | 2.37E-69 | 4.018484098 | up |
| aae-miR-12-3p | 2.46E-12 | -1.916482552 | down |
| aae-miR-12-5p | 2.24E-160 | -2.36870513 | down |
| aae-miR-2765 | 7.2E-33 | 2.189567236 | up |
| aae-miR-282-5p | 2.54E-08 | 1.600933508 | up |
| aae-miR-281-5p | 2.45E-52 | -2.292399364 | down |
| aae-miR-281-3p | 5.98E-67 | -2.851471676 | down |
| aae-miR-11900 | 3.04E-13 | -1.146146713 | down |
| aae-miR-92a-5p | 7.42E-12 | 1.666358971 | up |
| aae-miR-252-5p | 7.52E-27 | -2.266080937 | down |
| aae-miR-34-3p | 0.00000059 | 1.889991949 | up |
| aae-miR-11894a | 1.56E-18 | -1.415582026 | down |
| aae-let-7 | 1.52E-15 | 1.73698829 | up |
| aae-miR-1 | 0.0000148 | -1.085759047 | down |
| aae-miR-2946 | 2E-15 | 1.640833774 | up |
| aae-miR-1000 | 4.76E-30 | 3.113632919 | up |
| aae-miR-276-5p | 0.00000104 | 1.015643219 | up |
| aae-miR-11895 | 0.0000692 | 1.599820871 | up |
| aae-miR-11894b | 4.04E-09 | -1.434828335 | down |
| aae-miR-980-5p | 0.00000103 | 5.269839206 | up |
| aae-miR-2a-5p | 2.48E-08 | -1.754082556 | down |
| aae-miR-125-5p | 2.13E-09 | -2.15912571 | down |
| aae-miR-263a-5p | 0.00000015 | -2.21866546 | down |
| aae-miR-11916 | 0.00652452 | -1.202311098 | down |
| aae-miR-9a | 3.56E-48 | -4.793187169 | down |
| aae-miR-980-3p | 1.19E-10 | 4.340967365 | up |
| aae-miR-307 | 0.00040316 | 2.706179753 | up |
| aae-miR-252-3p | 0.00034254 | -1.319196039 | down |
| aae-miR-286a | 0.0000156 | 2.758815797 | up |
| aae-miR-308-3p | 0.00154086 | -1.516789281 | down |
| aae-miR-11921 | 0.00567318 | 3.094104885 | up |
| aae-miR-2944b-5p | 0.00192453 | 2.808791764 | up |
| aae-miR-316 | 0.00527684 | 1.722875408 | up |
| aae-miR-1175-5p | 5.34E-57 | -8.372343348 | down |
| aae-miR-375 | 0.00000038 | -3.757291336 | down |
| aae-miR-11893 | 0.00000086 | -3.380768661 | down |
| aae-miR-2942 | 0.02007577 | -5.623058887 | down |
| aae-miR-315-5p | 0.03383406 | -2.662300648 | down |
| aae-miR-124 | 0.00057955 | -7.186500488 | down |
| aae-miR-1174 | 5.09E-09 | -10.079528 | down |
| aae-miR-31 | 0.01134189 | -1.822655818 | down |
| aae-miR-1175-3p | 3.43E-20 | -7.381367557 | down |
