# Supplementary material for: Wolbachia mediates crosstalk between miRNA and Toll pathways to enhance resistance to dengue virus in Aedes aegypti
Source: PLoS Pathog. 2024 Jun 17;20(6):e1012296. doi: 10.1371/journal.ppat.1012296 (PMC11213346; doi:10.1371/journal.ppat.1012296)
Supplement: S3 Table — (DOCX) [file ppat.1012296.s003.docx]

**S3 Table. The sequence-specific reagents used in the miRNA and lncRNA function assays.**

| **RNA** | **Reagent name** | **Function assay** | **Sequence（5'-3'）** |
| --- | --- | --- | --- |
| aae-miR-34-3p | aae-miR-34-3p agomir | miRNA upregulation assay | CAACCACUAUCCGCCCUGCCGCC  CGGCAGGGCGGAUAGUGGUUGUU |
|  | agomir negative control | miRNA upregulation assay | UUCUCCGAACGUGUCACGUTT  ACGUGACACGUUCGGAGAATT |
| aae-miR-34-3p | aae-miR-34-3p antagomir | miRNA downregulation assay/  Function rescue assay | GGCGGCAGGGCGGAUAGUGGUUG |
|  | antagomir negative control | miRNA downregulation assay/  Function rescue assay | CAGUACUUUUGUGUAGUACAA |
| aae-lnc-2268 | aae-lnc-2268 siRNA | miRNA downregulation assay/  Function rescue assay | GCACACUUCUAGCGGGAAUTT  AUUCCCGCUAGAAGUGUGCTT |
|  | siRNA negative control | miRNA downregulation assay/  Function rescue assay | UUCUCCGAACGUGUCACGUTT  ACGUGACACGUUCGGAGAATT |
