# Supplementary material for: Wolbachia mediates crosstalk between miRNA and Toll pathways to enhance resistance to dengue virus in Aedes aegypti
Source: PLoS Pathog. 2024 Jun 17;20(6):e1012296. doi: 10.1371/journal.ppat.1012296 (PMC11213346; doi:10.1371/journal.ppat.1012296)
Supplement: S4 Table — (DOCX) [file ppat.1012296.s004.docx]

**S4 Table. The sequence of mRNA primers used in qPCR.**

| **Transcript ID** | **Primer Name** | **Sequence（5'-3'）** |
| --- | --- | --- |
| AAEL012410-RA | *AGO1B* forward | ATACTTCGCCGCCAAGGCCT |
|  | *AGO1B* reverse | TGTGGCACCAGCTGGAGGAG |
| AAEL001612-RA | *DCR-1* forward | TGGGGACTTCCGGCTAACGTG |
|  | *DCR-1* reverse | CACCGTCACCACTGCCGCTA |
| AAEL003841-RA | *DEFA* forward | CTATCAGGCTGCCGTGGAG |
|  | *DEFA* reverse | CAATGAGCAGCACAAGCACTATC |
| AAEL003832-RA | *DEFC* forward | TTGTTTGCTTCGTTGCTCTTT |
|  | *DEFC* reverse | ATCTCCTACACCGAACCCACT |
| AAEL003857-RA | *DEFD* forward | GGCGTTGGTGATAGTGCTTG′ |
|  | *DEFD* reverse | CACACCTTCTTGGAGTTGCAG |
| AAEL003849-RA | *DEFE* forward | GTGCGGGACACTGTCTAGCC |
|  | *DEFE* reverse | CAATCCTAATAATACTCATGTGCGG |
| AAEL000627-RA | *CECA* forward | CAGGTGGCCTTAAGAAGCTG |
|  | *CECA* reverse | GCTTTAGCCCCAGCTACAACT |
| AAEL004223-RA | *CECB* forward | GCTGAAGAAGCTGGGAAAAAAG |
|  | *CECB* reverse | CTTCCCAGTCCCTTGATGCC |
| AAEL000598-RA | *CECD* forward | ATGAACTTCACTAAGCTGTT |
|  | *CECD* reverse | TCATTTTCCAATCGCTTTTAT |
| AAEL000611-RA | *CECE* forward | GAAAGCACTTCCCGTAGTAACTG |
|  | *CECE* reverse | GTTAGTTATCACAATTCCCCCATG |
| AAEL000625-RA | *CECF* forward | GTGTTCAAAGCATCGGAAAAAG |
|  | *CECF* reverse | GCTGACATTCACAATCTATCTCCG |
| AAEL015515-RA | *CECG* forward | GTTATTTCTCCTGATCGCCG |
|  | *CECG* reverse | CTCGTTTTCCTGCACCTCCC |
| AAEL017211-RA | *CECH* forward | CTTCACCAAGCTGCTATTGGT |
|  | *CECH* reverse | AACTTTTTTGCCAATCTTCTTCAGC |
| AAEL000775-RA | *CECI* forward | GGCTATTCGTTTTCGTCATTTTC |
|  | *CECI* reverse | CTACGTTTTTGCCAGCCTTTTC |
| AAEL000777-RA | *CECJ* forward | GCTATTCGTTTTCGTCATTTTTG |
|  | *CECJ* reverse | CTTTTCAATCTTTTTGCCCAG |
| AAEL000621-RA | *CECN* forward | CGGCAAGAAATTGGAAAAAGTC |
|  | *CECN* reverse | GAATCGATCATCCTAGGGCC |
| AAEL007768-RA | *MyD88* forward | TGGTCAATTCTACGCCCGTC |
|  | *MyD88* reverse | ACTTGAAACAACCCTCGCCA |
| AAEL000032-RA | *RPS6* forward | GAAGTTGAACGTATCGTTTC |
|  | *RPS6* reverse | GAGATGGTCAGCGGTGATTT |
