# Supplementary material for: Wolbachia mediates crosstalk between miRNA and Toll pathways to enhance resistance to dengue virus in Aedes aegypti
Source: PLoS Pathog. 2024 Jun 17;20(6):e1012296. doi: 10.1371/journal.ppat.1012296 (PMC11213346; doi:10.1371/journal.ppat.1012296)
Supplement: S5 Table — (DOCX) [file ppat.1012296.s005.docx]

### S5 Table. The primer sequence used in quantification of noncoding RNAs via PCR.

| **Noncoding RNA** | **Primer name** | **Sequence（5'-3'）** |
| --- | --- | --- |
| aae-miR-999 | aae-miR-999 forward | ATACTTCGCCGCCAAGGCCT |
| aae-miR-980-5p | aae-miR-980-5p forward | CGGCCGTTCATTGGGTCATCTAGC |
| aae-miR-980-3p | aae-miR-980-3p forward | TAGCTGCCTAGTGAAGGGC |
| aae-miR-137 | aae-miR-137 forward | TATTGCTTGAGAATACACGTAG |
| aae-miR-1000 | aae-miR-1000 forward | ATATTGTCCTGTCACAGCAGT |
| aae-miR-11921 | aae-miR-11921 forward | AAATGGGACTGATATGCGAGTAT |
| aae-miR-277-3p | aae-miR-277-3p forward | TAAATGCACTATCTGGTACGAC |
| aae-miR-2944b-5p | aae-miR-2944b-5p forward | GAAGGAACTCCCGGTGTGATATA |
| aae-miR-252-5p | aae-miR-252-5p forward | TAAGTACTAGTGCCGCAGGAG |
| aae-miR-286a | aae-miR-286a forward | GACTAGACCGAACACTCGCGTCCT |
| aae-miR-989 | aae-miR-989 forward | TGTGATGTGACGTAGTGGTAC |
| aae-miR-2765 | aae-miR-2765 forward | TGGTAACTCCACCACCGTTGGC |
| aae-miR-281-5p | aae-miR-281-5p forward | AAGAGAGCTATCCGTCGAC |
| aae-let-7 | aae-let-7 forward | TGAGGTAGTTGGTTGTATAGT |
| aae-miR-12-5p | aae-miR-12-5p forward | TGAGTATTACATCAGGTACTGGT |
| aae-miR-2a-3p | aae-miR-2a-3p forward | TATCACAGCCAGCTTTGAAGAGC |
| aae-miR-281-3p | aae-miR-281-3p forward | TGTCATGGAATTGCTCTCTTTA |
| aae-miR-11893 | aae-miR-11893 forward | TTCCTGACTTATACGCTTACCT |
| aae-miR-375 | aae-miR-375 forward | TTTGTTCGTTTGGCTCGAGTTA |
| aae-miR-9a | aae-miR-9a forward | TCTTTGGTTATCTAGCTGTATGA |
| aae-miR-2942 | aae-miR-2942 forward | TATTCGAGACTTCACGAGTTAAT |
| aae-miR-124 | aae-miR-124 forward | TAAGGCACGCGGTGAATGC |
| aae-miR-1175-5p | aae-miR-1175-5p forward | AAGTGGAGTAGTGGTCTCATCG |
| aae-miR-34-3p | aae-miR-34-3p forward | CAACCACTATCCGCCCTGCCGCC |
| aae-miR-307 | aae-miR-307 forward | CACAACCTCCTTGAGTGAGCGA |
| aae-miR-87 | aae-miR-87 forward | GTGAGCAAATTTTCAGGTGTGT |
| aae-miR-263a-5p | aae-miR-263a-5p forward | AATGGCACTGGAAGAATTCACGG |
| aae-miR-315-5p | aae-miR-315-5p forward | TTTTGATTGTTGCTCAGAAAGC |
| aae-miR-1175-3p | aae-miR-1175-3p forward | TGAGATTCTACTTCTCCGACTTAA |
| aae-miR-1174 | aae-miR-1174 forward | TCAGATCTACTTAATACCCAT |
| aae-lnc-2268 | aae-lnc-2268 forward | GTAGCCGTGATCCTCGTCAG |
| aae-lnc-2268 | aae-lnc-2268 reverse | AGGAACAAAACTTCTTGTGAACCA |
| aae-*U6* | aae-*U6* forward | CACGCAAAATCGTGAAGCG |
| aae-*actin-5C* | aae-*actin-5C* forward | CGTTCGTGACATCAAGGAAA |
| aae-*actin-5C* | aae-*actin-5C* reverse | GAACGATGGCTGGAAGAGAG |
| aae-*5S rRNA* | aae-*5S rRNA* forward | CGCGTCAGAATGTGAACTGC |
| aae-*7S* | aae-*7S* forward | TCAGTGTACAAGAAGCTGACCGGA |
| aae-*7S* | aae-*7S* reverse | TTCCGCGCGCGCTCACTTATTAGATT |
