# Supplementary material for: Wolbachia mediates crosstalk between miRNA and Toll pathways to enhance resistance to dengue virus in Aedes aegypti
Source: PLoS Pathog. 2024 Jun 17;20(6):e1012296. doi: 10.1371/journal.ppat.1012296 (PMC11213346; doi:10.1371/journal.ppat.1012296)
Supplement: S6 Table — (DOCX) [file ppat.1012296.s006.docx]

**S6 Table. The fluorescent RNA probes used in FISH.**

| **Probe name** | **Sequence（5'-3'）** |
| --- | --- |
| FAM-aae-miR-34-3p | GGCGGCAGGGCGGATAGTGGTTG |
| Cy3-*Wolbachia* 16s rRNA | GATGTCTTCACCAGAGCAAA |
| Cy3-aae-lnc-2268 | CTTCTGTGAGTACCGTCATTATC |
