# Supplementary figures and images for: Wolbachia mediates crosstalk between miRNA and Toll pathways to enhance resistance to dengue virus in Aedes aegypti
Source: PLoS Pathog. 2024 Jun 17;20(6):e1012296. doi: 10.1371/journal.ppat.1012296 (PMC11213346; doi:10.1371/journal.ppat.1012296)

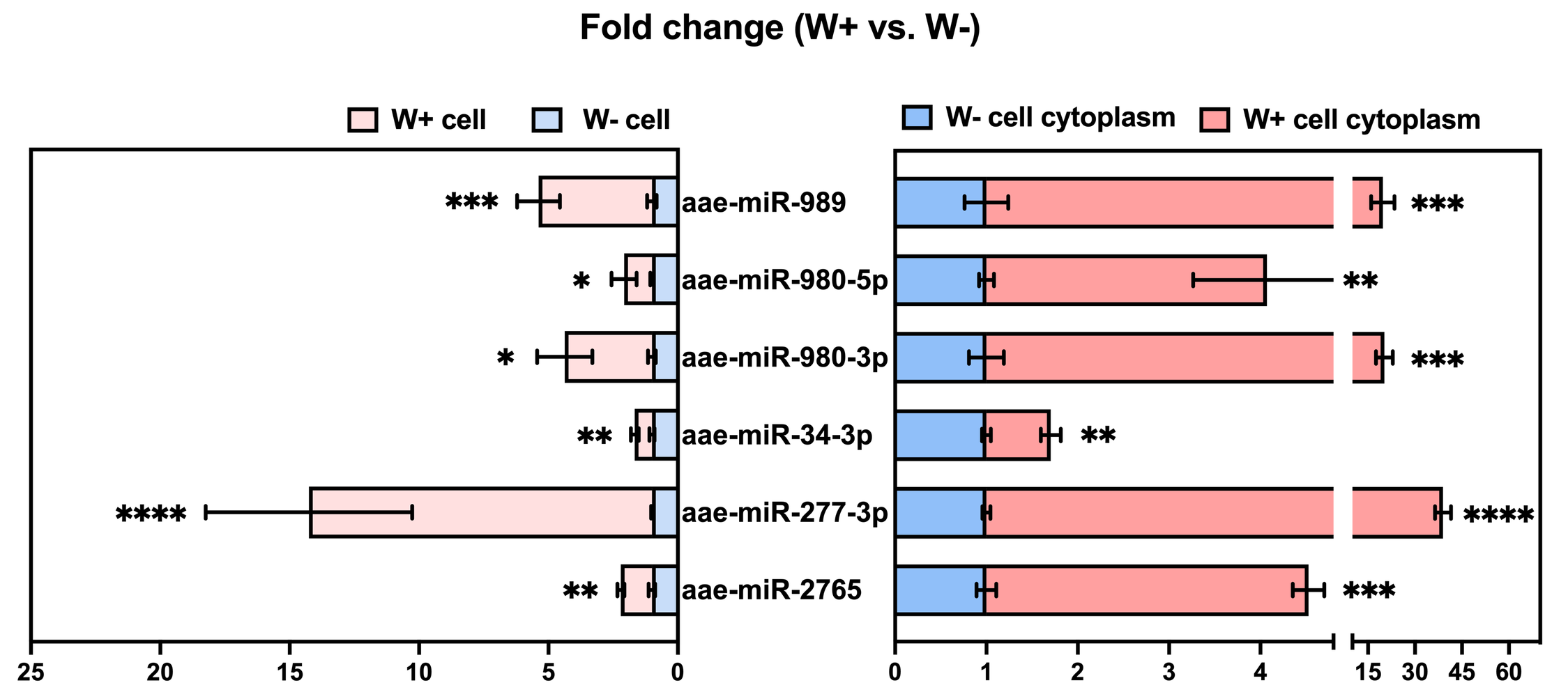

Supplement: S1 Fig — The expression of 6 out of the top 15 up-regulated DE miRNAs was increased in whole-cell samples (left panel, two-sided t test, W-: n = 4, W+: n = 4, aae-miR-989: P = 0.0005, aae-miR-980-5p: P = 0.0411, aae-miR-980-3p: P = 0.0156, aae-miR-34-3p: P = 0.0092, aae-miR-277-3p: P = 8.4345×10^−5^, aae-miR-2765: P = 0.0025) and cytoplasm samples (right panel, two-sided t test, W-: n = 3, W+: n = 3, aae-miR-989: P = 0.0005, aae-miR-980-5p: P = 0.0043, aae-miR-980-3p: P = 0.0002, aae-miR-34-3p: P = 0.0026, aae-miR-277-3p: P = 1.3807×10^−6^, aae-miR-2765: P = 0.0002) from W+ cells in qPCR analysis. The expression of miRNAs was normalized to the amount of cDNA template (100 ng). The error bars indicate the standard error. ****P < 0.0001; ***P < 0.001; **P < 0.01; *P < 0.05. (TIF) [file ppat.1012296.s007.tif]

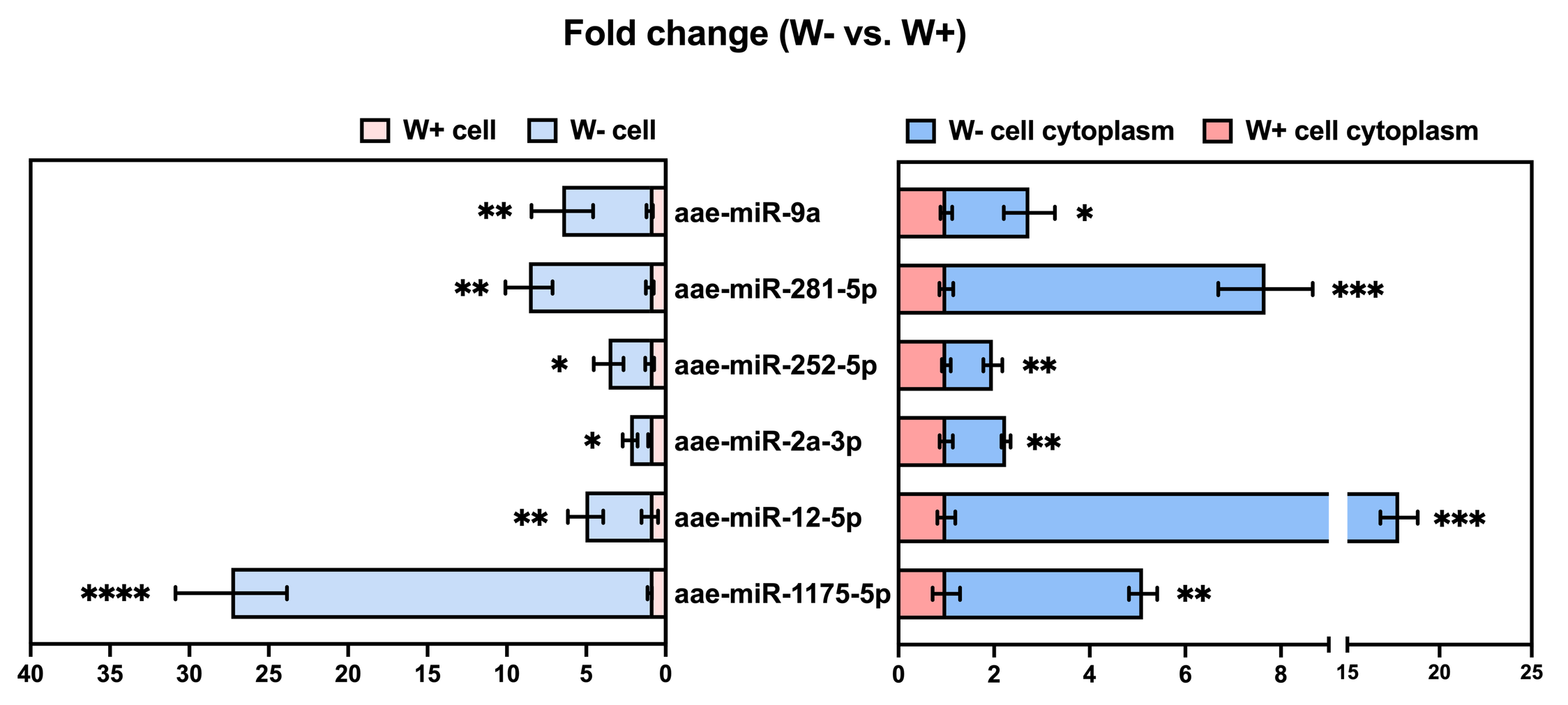

Supplement: S2 Fig — qPCR analysis showed that the expression of 6 out of the top 15 downregulated DE miRNAs was decreased in whole-cell samples (left panel, two-sided t test, W-: n = 4, W+: n = 4, aae-miR-9a: P = 0.0043, aae-miR-281-5p: P = 0.0010, aae-miR-252-5p: P = 0.0449, aae-miR-2a-3p: P = 0.0192, aae-miR-12-5p: P = 0.0089, aae-miR-1175-5p: P = 2.1435×10^−6^) and cytoplasm samples (right panel, two-sided t test, W-: n = 3, W+: n = 3, aae-miR-9a: P = 0.0130, aae-miR-281-5p: P = 0.0005, aae-miR-252-5p: P = 0.0087, aae-miR-2a-3p: P = 0.0058, aae-miR-12-5p: P = 0.0002, aae-miR-1175-5p: P = 0.0033) from W+ cells in small RNA sequencing. The expression of miRNAs was normalized to the amount of cDNA template (100 ng). The error bars indicate the standard error. ****P < 0.0001; ***P < 0.001; **P < 0.01; *P < 0.05. (TIF) [file ppat.1012296.s008.tif]

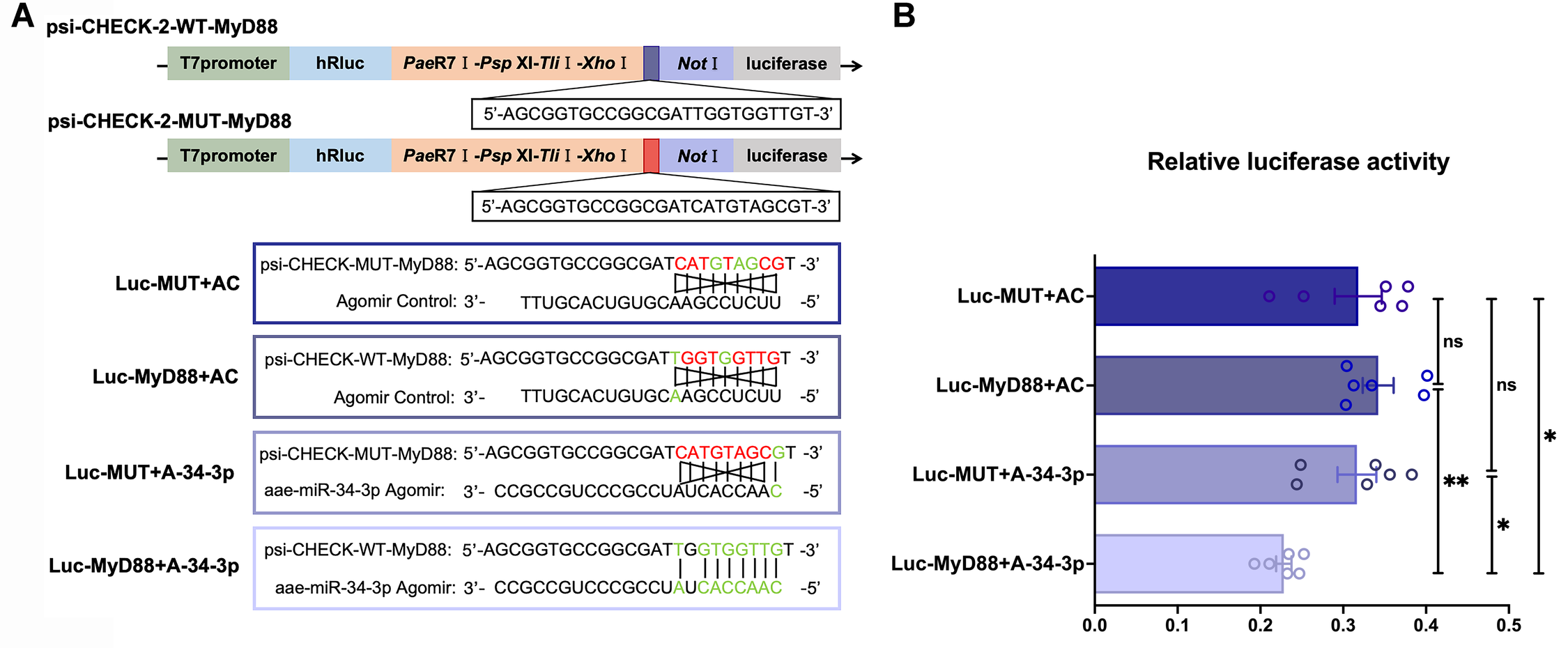

Supplement: S3 Fig — (A) Schematic representation of the construct psi-CHECK-2 plasmids used in the dual luciferase reporter assay shown in the upper panel. A schematic diagram of the predicted binding sites between aae-miR-34-3p agomir and MyD88 plasmids in the dual luciferase reporter assay shown in the bottom panel. (B) The binding relationship between MyD88 and aae-miR-34-3p was determined via a dual-luciferase reporter assay (one-way ANOVA, Luc-MUT+AC: n = 6, Luc-MyD88+AC: n = 6, Luc-MUT+A-34-3p: n = 6, Luc-MyD88+A-34-3p: n = 6, Luc-MUT+AC vs. Luc-MyD88+A-34-3p: P = 0.0326, Luc-MUT+AC vs. Luc-MyD88+AC: P = 0.8503, Luc-MUT+AC vs. Luc-MUT+A-34-3p: P = 0.9680, Luc-MyD88+AC vs. Luc-MyD88+A-34-3p:P = 0.0055, Luc-MUT+A-34-3p vs. Luc-MyD88+A-34-3p: P = 0.0363). Luc-MUT+AC: group cotransfected with psi-CHECK-2-MUT-MyD88 and agomir control, Luc-MyD88+A-34-3p: group with cotransfection of psi-CHECK-2-WT-MyD88 and aae-miR-34-3p agomir, Luc-MyD88+AC: group cotransfected with psi-CHECK-2-WT-MyD88 and agomir control, Luc-MUT+A-34-3p: group cotransfected with psi-CHECK-2-MUT-MyD88 and aae-miR-34-3p agomir. The error bars indicate the standard error. Each circle indicates a replicate per tested group. **P < 0.01; *P < 0.05; ns, non-significant. (TIF) [file ppat.1012296.s009.tif]

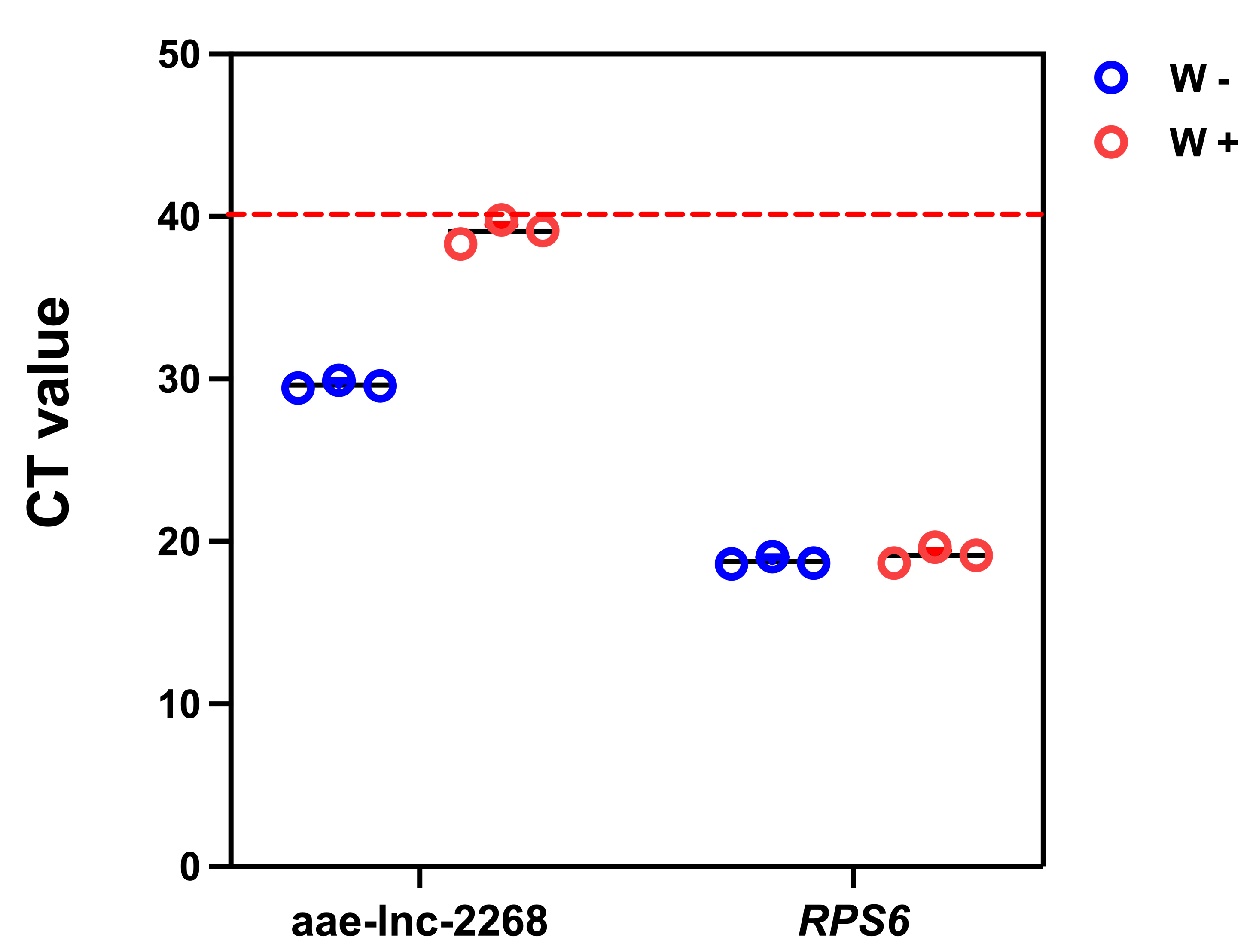

Supplement: S4 Fig — The black line indicates the mean Ct value. Each circle indicates a Ct value for aae-lnc-2268 or the RPS6 gene. The red dashed line indicates the threshold Ct value of 40. (TIF) [file ppat.1012296.s010.tif]
