# Supplementary material for: Wolbachia mediates crosstalk between miRNA and Toll pathways to enhance resistance to dengue virus in Aedes aegypti
Source: PLoS Pathog. 2024 Jun 17;20(6):e1012296. doi: 10.1371/journal.ppat.1012296 (PMC11213346; doi:10.1371/journal.ppat.1012296)

Figure 3A

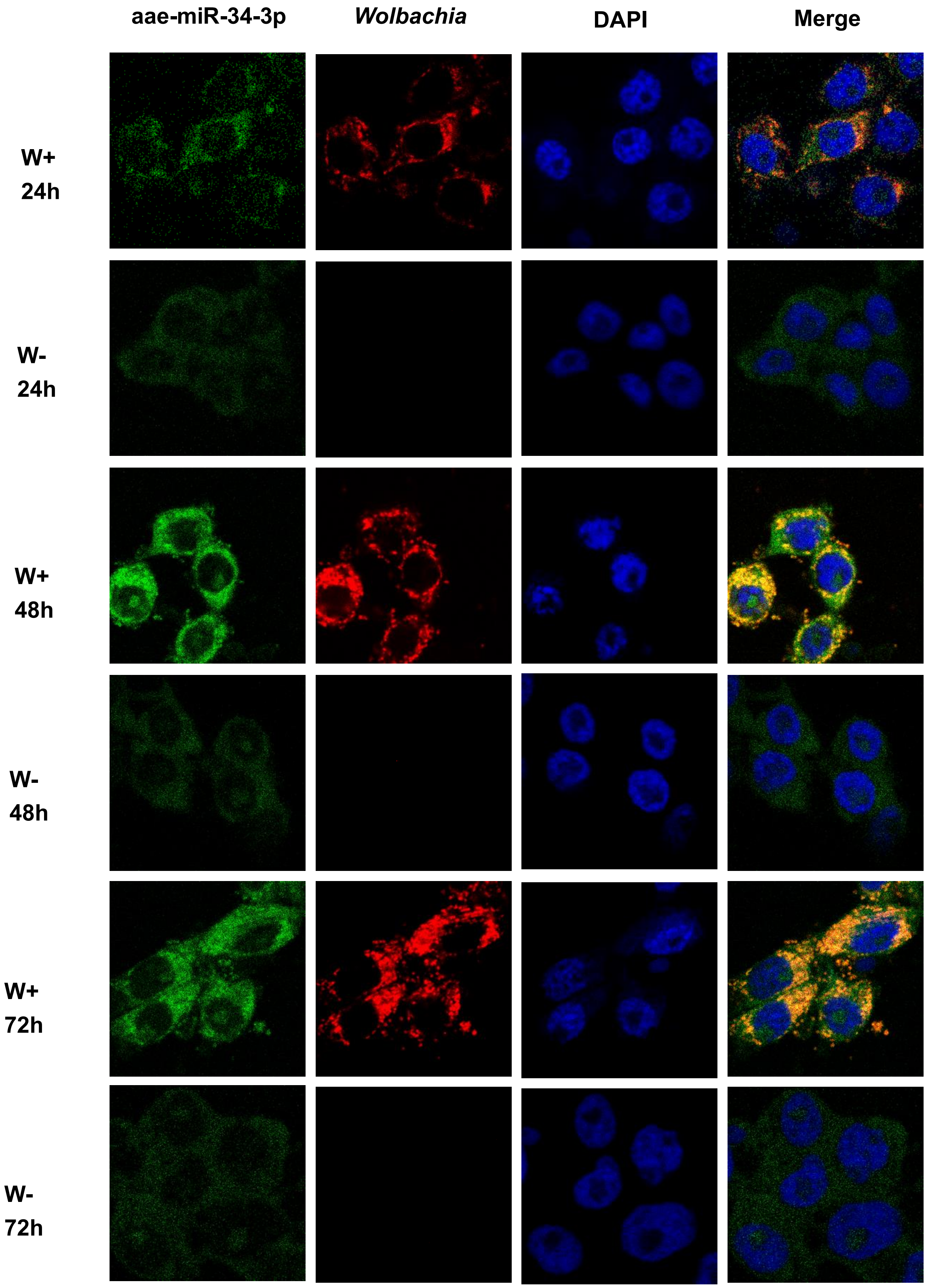
aae-miR-34-3p
Wolbachia
DAPI
Merge
W+
24h
W-
24h
W+
48h
W-
48h
W+
72h
W-
72h

Figure 3F

The following image depicts the MyD88 band, captured using a long exposure time to ensure optimal clarity (From left to right are marker, W-, and W+ cells).

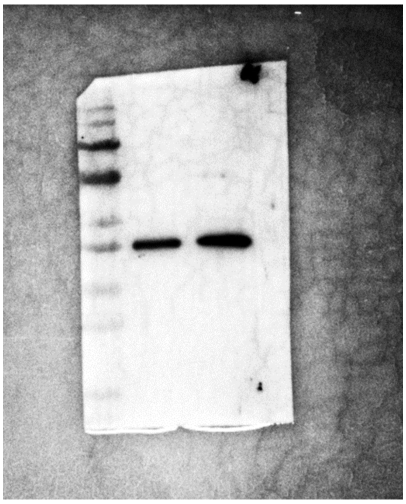

The image below illustrates the β-actin band, which was captured using a short exposure time (From left to right are marker, W-, and W+ cells).

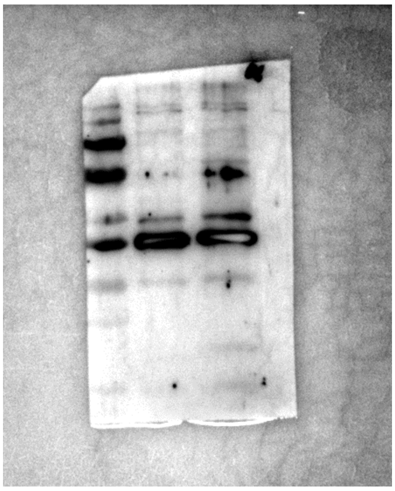

Figure 3G

S1

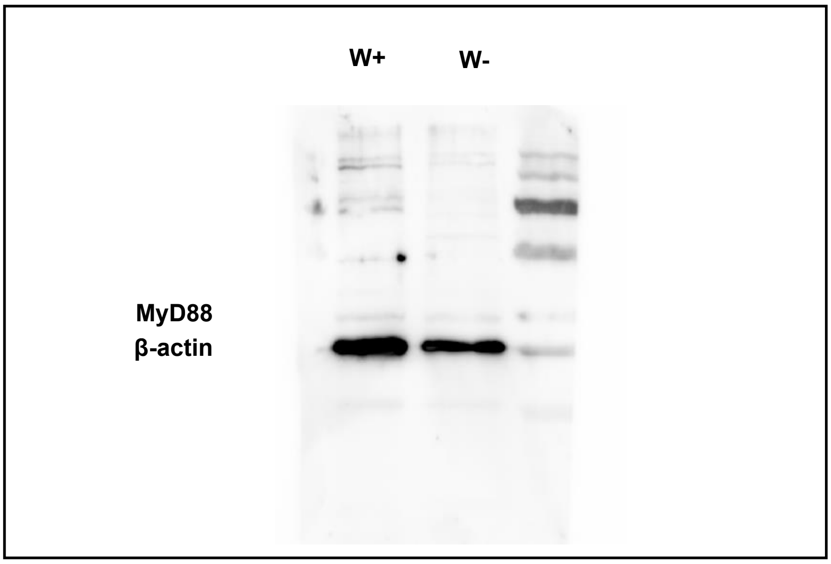

S2

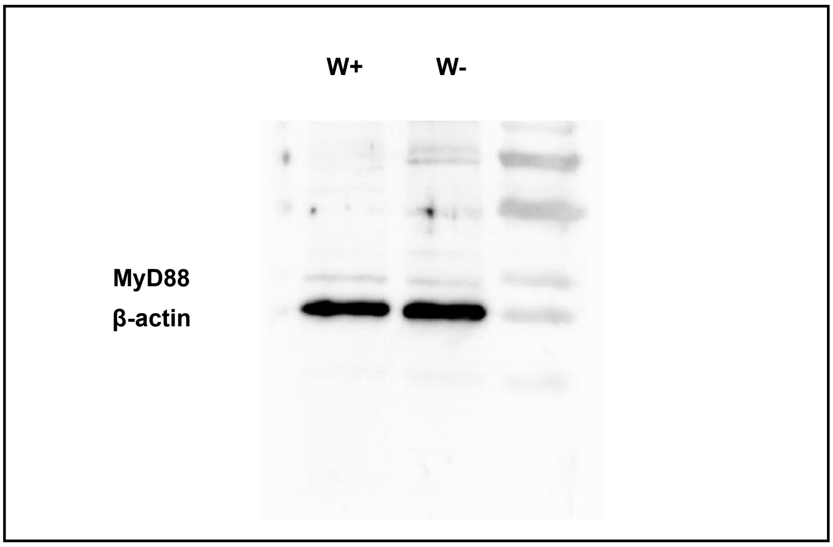

S3

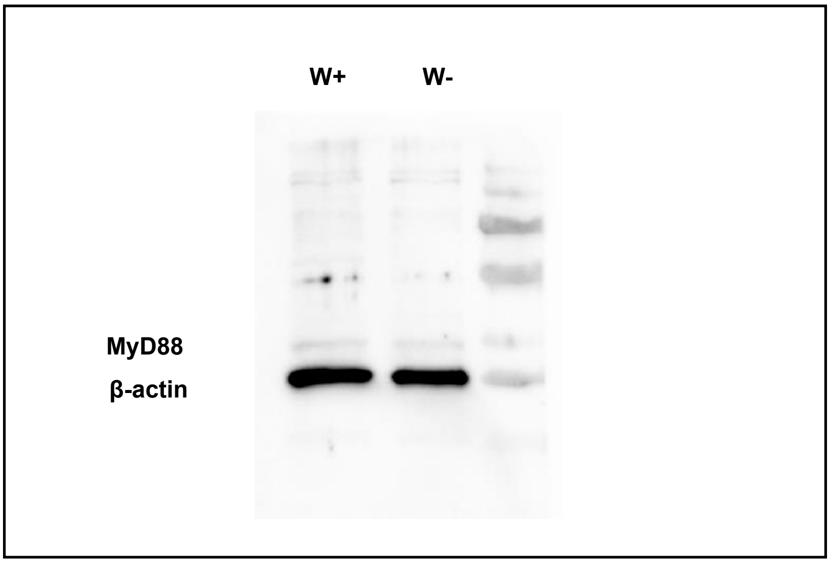

Figure 3I

The chemiluminescence image demonstrates the presence of the β-actin band under a short exposure time (From left to right are AC, A-34-3p, AC, and A-34-3p).

The image was captured using Chemiluminescence to demonstrate the presence of the MyD88 band under a long exposure time (From left to right are AC, A-34-3p, AC, and A-34-3p).

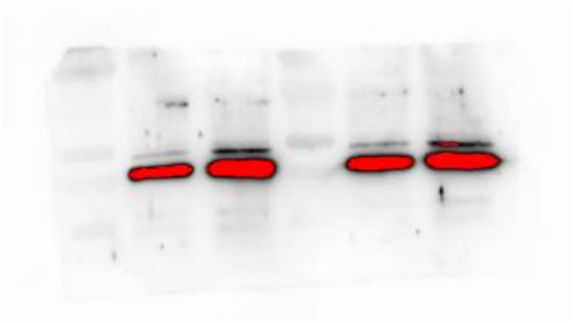

The visible light image is utilized to demonstrate the protein marker in colorimetric detection.

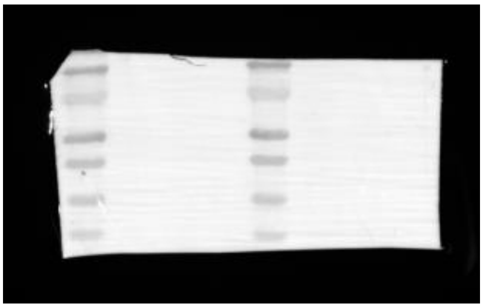

The merged image demonstrates the colocalization of MyD88, β-actin, and the protein marker bands (From left to right are marker, AC, A-34-3p, marker, AC, and A-34-3p).

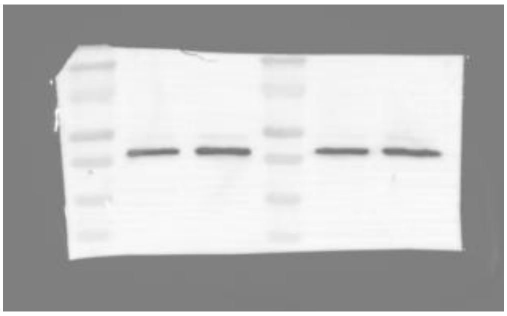

Figure 3J

S1

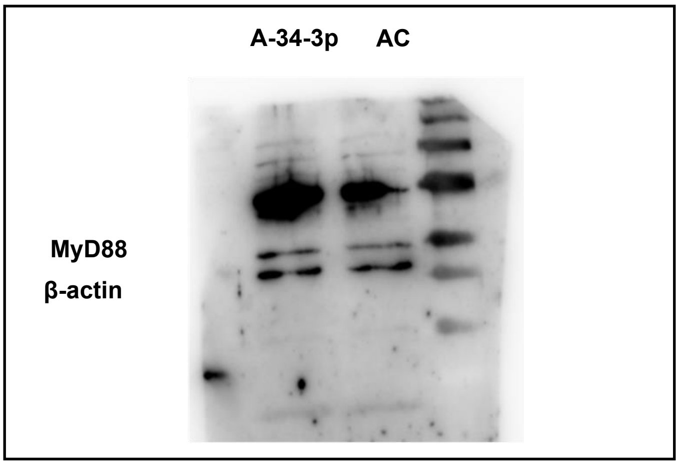

S2

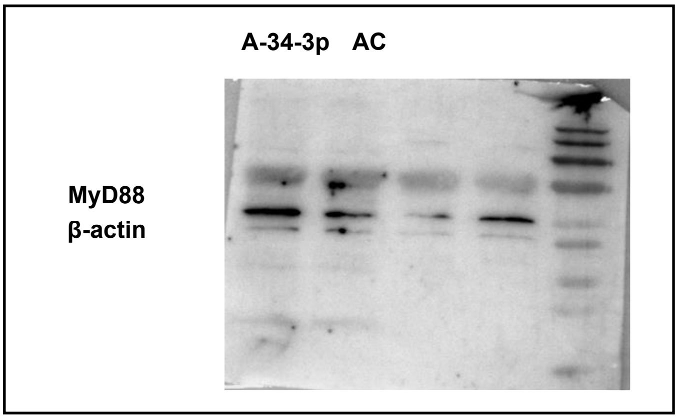

S3

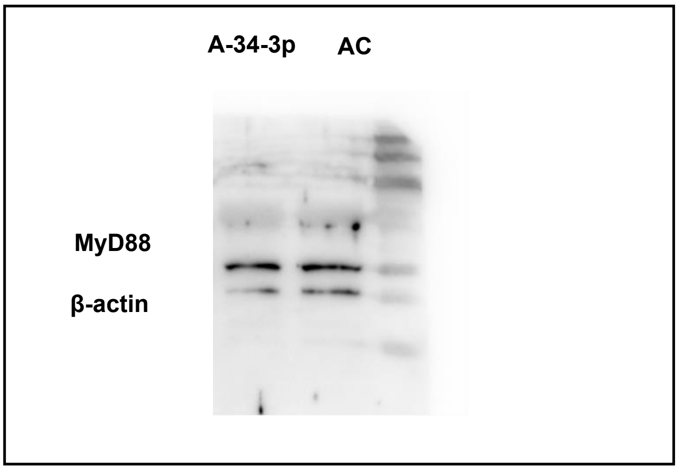

S4

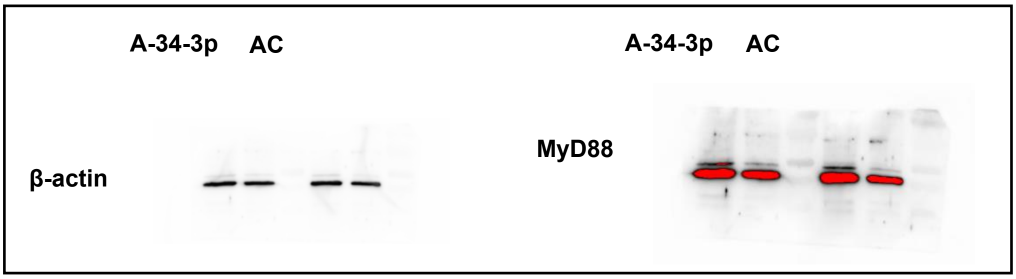

Figure 3L

The chemiluminescence image demonstrates the presence of MyD88 and β-actin bands (From left to right are ATC, AT-34-3p, ATC, and AT-34-3p).

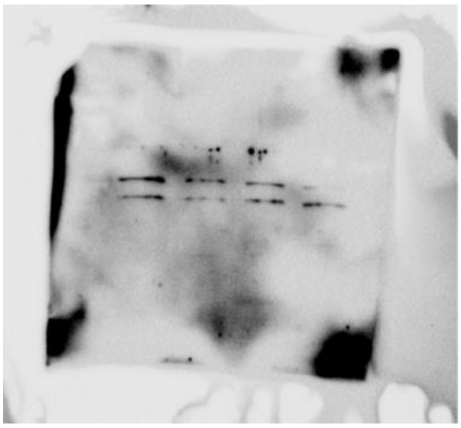

The visible light image is utilized to demonstrate the protein marker in colorimetric detection.

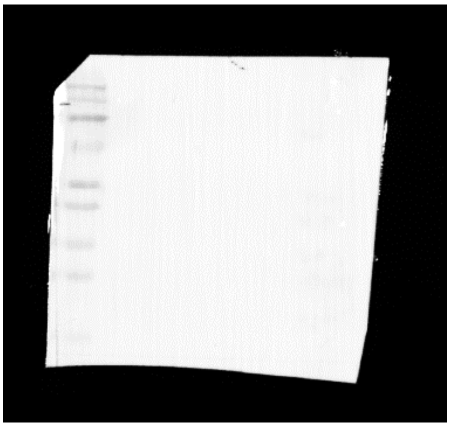

The merged image demonstrates the colocalization of MyD88, β-actin, and the protein marker bands (From left to right are marker, ATC, AT-34-3p, ATC, and AT-34-3p).

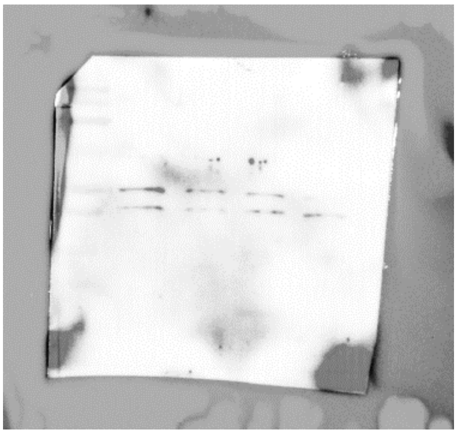

Figure 3M

S1-S2

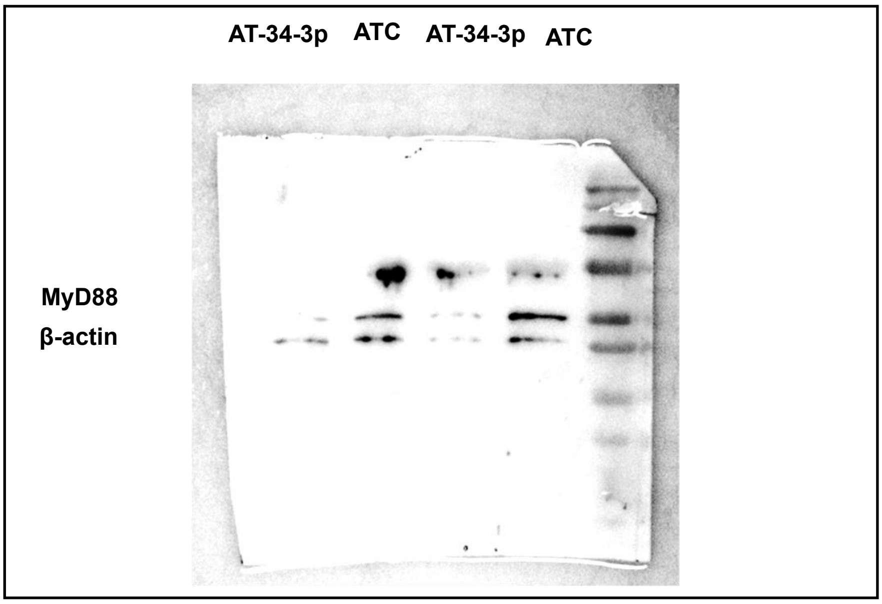

S3

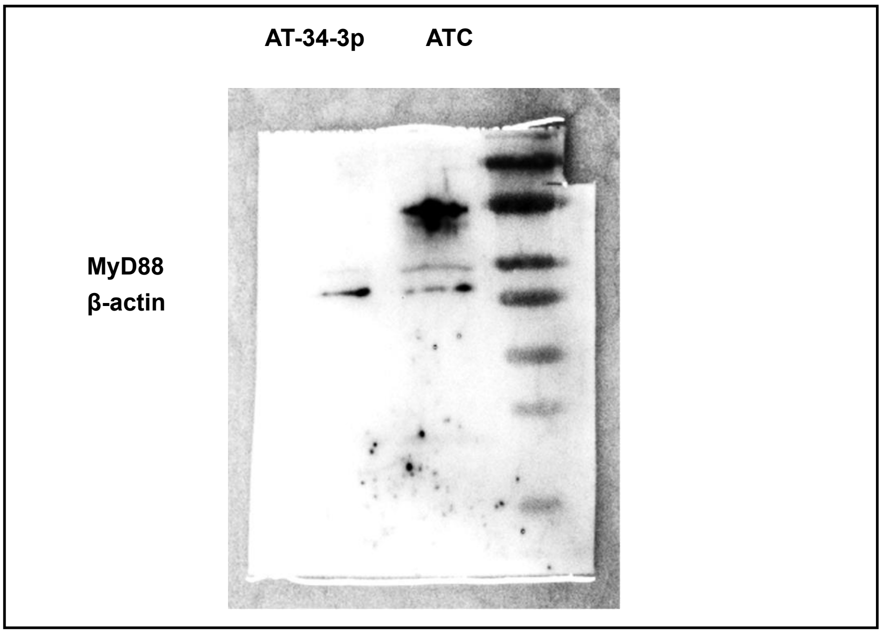

S4

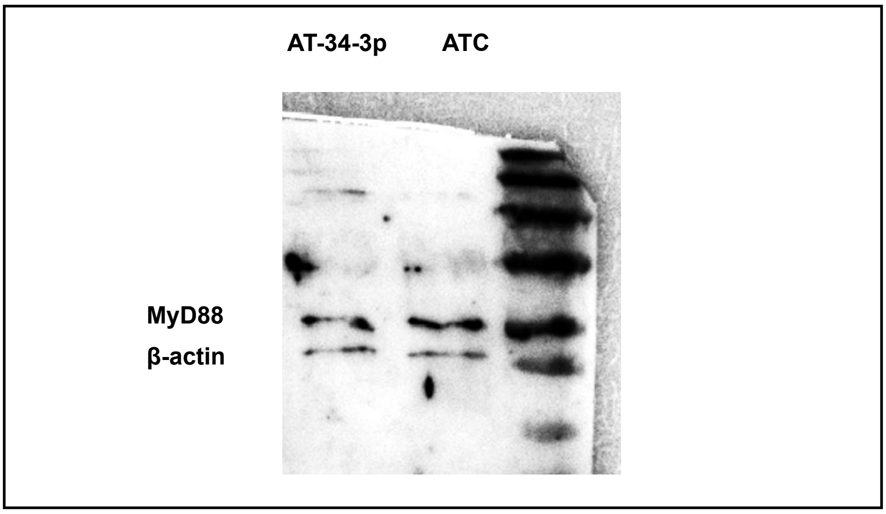

Figure 4C

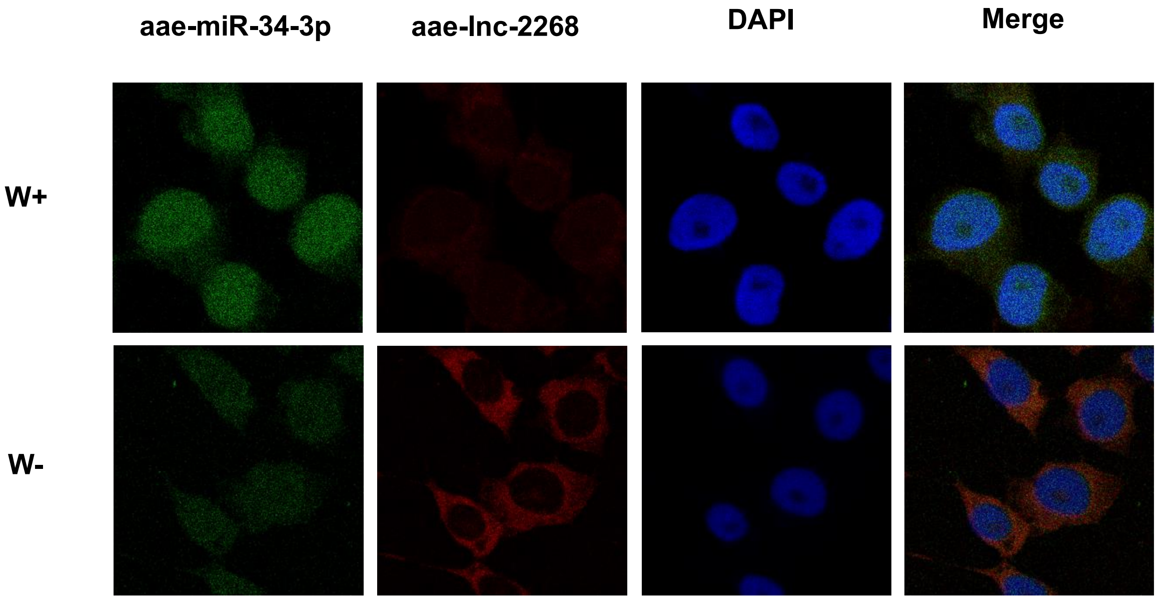

Figure 4D

aae-lnc-2268 RPS6

W+ W- W+ W-

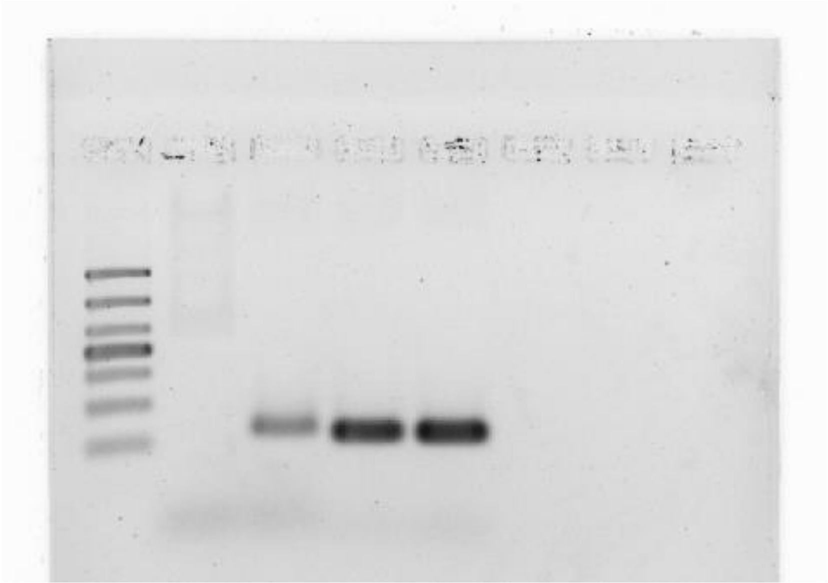

Supplement: S1 Raw Images — (PDF) [file ppat.1012296.s011.pdf]
